# Supplementary material for: Comparative Proteomics Unveils LRRFIP1 as a New Player in the DAPK1 Interactome of Neurons Exposed to Oxygen and Glucose Deprivation
Source: Antioxidants (Basel). 2020 Nov 30;9(12):1202. doi: 10.3390/antiox9121202 (PMC7761126; doi:10.3390/antiox9121202)
Supplement: Supplementary file 1 [file antioxidants-09-01202-s001.zip › Table S1.pdf]

Supplementary material of the MS **'Comparative proteomics unveils LRRFIP1 as a new player in the DAPK1 interactome of neurons exposed to oxygen and glucose deprivation'**, by DeGregorio-Rocasolano et al.

Table S1. Protein partners in the DAPK1 interactome of control neurons (abbreviations: m: mouse; r: rat; Cp: Cavia porcellus; Cla: Chinchilla lanigera; Cg: Cricetulus griseus; Clo: Cricetulus longicaudatus; Hg: Heterocephalus glaber; Ma: Mesocricetus aurus; Mg: Myodes glareolus; Sc: Spermophilus citellus; St: Spermophilus tridecemlineatus; Tm: Tokudaia muenninki).

Accession	Gene symbol	Description	Species	ΣCoverage	Σ# Proteins	Σ# Unique Peptides	Σ# Peptides	Σ# PSMs	# AAs	MW [kDa]	calc. pI
Q6QN02	-	Homeobox prox 1 (Fragment)	Cla	17.2	5	2	2	3	145	16.0	6.19
Q9CPN9	*2210010C04Rik*	RIKEN cDNA 2210010C04 gene (trypsinogen 7)	m	4.9	1	1	1	2	247	26.4	7.93
B2RYJ4	*Aasdhppt*	L-aminoadipate-semialdehyde dehydrogenase-phosphopantetheinyl transferase	r	4.9	1	1	1	2	309	35.8	6.65
B7ZCU1	*Abi1*	Abl-interactor 1	m	4.1	11	1	1	1	388	42.5	6.20
Q8K4G5	*Ablim1*	Actin-binding LIM protein 1	m	15.6	1	12	12	23	861	96.7	8.63
Q6KC51	*Ablim2*	Actin-binding LIM protein 2	r	3.8	2	2	2	3	612	68.0	7.94
Q3TS02	*Acly*	ATP citrate lyase	m	1.6	6	1	1	1	491	53.7	7.06
Q99KI0	*Aco2*	Aconitate hydratase, mitochondrial	m	1.9	2	1	1	1	780	85.4	7.93
E5F0Z2	*Actb*	Beta-actin (Fragment)	Mg	69.7	4	1	22	1721	353	39.1	5.69
P48975	*Actb*	Actin, cytoplasmic 1	Cg	69.3	12	1	20	1086	375	41.7	5.39
Q8BFZ3	*Actbl2*	Beta-actin-like protein 2	m	31.1	3	2	9	717	376	42.0	5.49
P68033	*Actc1*	Actin, alpha cardiac muscle 1	m	45.1	6	4	15	771	377	42.0	5.39
Q6GMN8	*Actn1*	Alpha-actinin-1	r	15.2	7	4	12	38	887	102.5	5.48
Q9JI91	*Actn2*	Alpha-actinin-2	m	8.4	3	3	7	26	894	103.8	5.45
Q9QXQ0	*Actn4*	Alpha-actinin-4	r	27.6	4	11	19	69	911	104.8	5.44
Q5M9F7	*Actr10*	ARP10 actin-related protein 10 homolog (S. cerevisiae)	r	5.5	2	2	2	2	417	46.2	7.37
P61164	*Actr1a*	Alpha-centractin	m	6.7	2	2	2	10	376	42.6	6.64
P61161	*Actr2*	Actin-related protein 2	m	16.2	2	6	6	29	394	44.7	6.74
Q99JY9	*Actr3*	Actin-related protein 3	m	18.4	3	7	7	17	418	47.3	5.88
Q63028	*Add1*	Alpha-adducin	r	24.9	3	13	14	63	735	80.3	6.15
Q05764	*Add2*	Beta-adducin	r	17.9	2	10	11	44	725	80.5	5.97
G3V9D7	*Add3*	Adducin 3 (Gamma), isoform CRA_a	r	11.5	2	1	7	11	705	78.8	5.95

Q9JMB5	*Adrm1*	Proteasomal ubiquitin receptor ADRM1	r	3.9	1	1	1	4	407	42.1	5.07
G3V6Z3	*Afap1*	Actin filament-associated protein 1	r	7.7	3	4	4	8	731	80.7	8.68
Q3UHD9	*Agap2*	Arf-GAP with GTPase, ANK repeat and PH domain-containing protein 2	m	3.5	2	3	3	4	1186	124.4	9.89
Q70FJ1	*Akap9*	A-kinase anchor protein 9	m	2.7	2	9	9	13	3797	435.9	5.03
Q9JHE0	*Akap9*	A-kinase anchor protein 9 (Fragment)	r	2.3	1	1	1	1	395	45.1	4.64
O70473	*Akr1a1*	Alcohol dehydrogenase [NADP(+)] (Fragment)	Cg	8.3	3	1	1	2	228	25.4	6.28
Q6WDN9	*Alb*	Preproalbumin	Cp	3.8	2	1	2	7	608	68.8	6.61
P05064	*Aldoa*	Fructose-bisphosphate aldolase A	m	14.0	9	4	4	5	364	39.3	8.09
P05063	*Aldoc*	Fructose-bisphosphate aldolase C	m	16.3	2	5	5	8	363	39.4	7.12
G3V6U3	*Alg2*	Asparagine-linked glycosylation 2 homolog (Yeast, alpha-1,3-mannosyltransferase), isoform CRA_a	r	2.2	1	1	1	1	415	47.3	7.97
D3ZXH7	*Alyref*	Aly/REF export factor	r	11.1	5	2	2	2	189	19.9	9.86
B1AXE8	*Amot*	Angiomotin (Fragment)	m	1.3	2	1	1	10	671	75.6	7.94
Q8C8R3	*Ank2*	Ankyrin-2	m	9.2	3	28	29	105	3898	426.0	5.17
D4A9E7	*Ankrd5*	Ankyrin repeat domain 5 (Predicted)	r	1.2	2	1	1	1	775	86.8	8.02
D3ZUY8	*Ap2a1*	Adaptor protein complex AP-2, alpha 1 subunit (Predicted)	r	10.6	2	4	8	13	977	107.6	7.06
P17427	*Ap2a2*	AP-2 complex subunit alpha-2	m	11.5	3	9	10	16	938	104.0	6.93
P62944	*Ap2b1*	AP-2 complex subunit beta	r	17.7	9	14	14	37	937	104.5	5.38
Q3TWV4	*Ap2m1*	AP-2 complex subunit mu	m	13.6	2	6	6	12	433	49.4	9.54
P62743	*Ap2s1*	AP-2 complex subunit sigma	m	12.0	1	2	2	2	142	17.0	6.18
G3V8Q9	*Apc*	Adenomatosis polyposis coli, isoform CRA_a	r	1.1	2	2	2	5	2842	310.3	7.37
P08226	*Apoe*	Apolipoprotein E	m	10.6	5	3	3	5	311	35.8	5.68
B4F779	*Appl2*	Adaptor protein, phosphotyrosine interaction, PH domain and leucine zipper containing 2	r	10.1	2	5	5	7	662	74.1	5.12
P61205	*Arf3*	ADP-ribosylation factor 3	m	32.0	4	3	5	20	181	20.6	7.43
P61750	*Arf4*	ADP-ribosylation factor 4	m	20.0	1	2	3	16	180	20.4	7.14
P84084	*Arf5*	ADP-ribosylation factor 5	m	25.6	1	2	4	18	180	20.5	6.79
Q6DFV3	*Arhgap21*	Rho GTPase-activating protein 21	m	1.3	3	2	2	3	1944	215.6	7.64
O55043	*Arhgef7*	Rho guanine nucleotide exchange factor 7	r	1.6	3	1	1	1	646	73.1	7.15
Q8VEH3	*Arl8a*	ADP-ribosylation factor-like protein 8A	m	14.5	2	3	3	11	186	21.4	7.77
Q99PD4	*Arpc1a*	Actin-related protein 2/3 complex subunit 1A	r	21.4	2	6	6	21	370	41.6	8.18
D3ZZU0	*Arpc2*	Actin related protein 2/3 complex, subunit 2 (Predicted), isoform CRA_a	r	17.7	4	5	5	14	300	34.3	7.36

B2GV73	*Arpc3*	Actin related protein 2/3 complex, subunit 3	r	13.5	2	2	2	4	178	20.5	8.60
P59999	*Arpc4*	Actin-related protein 2/3 complex subunit 4	m	16.1	1	3	3	18	168	19.7	8.43
Q4KLF8	*Arpc5*	Actin-related protein 2/3 complex subunit 5	r	20.5	4	2	2	5	151	16.3	5.67
A1L108	*Arpc5l*	Actin-related protein 2/3 complex subunit 5-like protein	r	8.5	4	1	1	4	153	17.0	6.80
Q7SIG6	*Asap2*	Arf-GAP with SH3 domain, ANK repeat and PH domain-containing protein 2	m	0.8	1	1	1	2	958	106.7	6.65
B8JJ76	*Atat1*	Alpha tubulin acetyltransferase 1	m	8.4	6	2	2	3	333	37.5	10.10
Q1M168	*Atcay*	Caytaxin	r	13.4	2	4	4	10	372	42.1	4.65
Q6PIC6	*Atp1a3*	Sodium/potassium-transporting ATPase subunit alpha-3	m	5.8	7	2	4	6	1013	111.6	5.41
P11507	*Atp2a2*	Sarcoplasmic/endoplasmic reticulum calcium ATPase 2	r	3.4	3	3	3	3	1043	114.7	5.34
Q8K314	*Atp2b1*	Atp2b1 protein (Fragment)	m	1.2	11	1	1	2	914	101.6	6.95
P15999	*Atp5a1*	ATP synthase subunit alpha, mitochondrial	r	20.1	3	9	9	27	553	59.7	9.19
G3V6D3	*Atp5b*	ATP synthase subunit beta	r	20.2	4	8	8	34	529	56.3	5.27
G3V7Y3	*Atp5d*	ATP synthase subunit delta, mitochondrial	r	13.7	3	2	2	6	168	17.6	5.24
P19511	*Atp5f1*	ATP synthase subunit b, mitochondrial	r	4.7	1	1	1	4	256	28.9	9.36
P31399	*Atp5h*	ATP synthase subunit d, mitochondrial	r	7.5	1	1	1	2	161	18.8	6.60
Q06647	*Atp5o*	ATP synthase subunit O, mitochondrial	r	5.6	2	1	1	1	213	23.4	10.02
A2A5A2	*Atp6v0a1*	ATPase, H+ transporting, lysosomal V0 subunit A1, isoform CRA_a	Hg	3.3	5	2	2	2	832	95.6	6.77
P51863	*Atp6v0d1*	V-type proton ATPase subunit d 1	m	6.6	1	2	2	5	351	40.3	5.00
D4A133	*Atp6v1a*	ATPase H+ transporting V1 subunit A	r	7.6	3	4	4	8	617	68.2	5.58
P62814	*Atp6v1b2*	V-type proton ATPase subunit B, brain isoform	m	9.4	3	4	4	11	511	56.5	5.81
D3ZW96	*Atp6v1h*	ATPase H+ transporting V1 subunit H	r	2.7	2	1	1	2	483	55.8	6.61
Q5U2U8	*Bag3*	Bcl2-associated athanogene 3	r	3.0	1	1	1	5	574	61.5	7.03
B1AZ45	*Baiap2*	Brain-specific angiogenesis inhibitor 1-associated protein 2	m	7.7	7	3	3	3	482	53.2	8.98
Q05175	*Basp1*	Brain acid soluble protein 1	r	35.5	1	2	4	18	220	21.8	4.51
Q91XV3	*Basp1*	Brain acid soluble protein 1	m	22.1	1	1	3	24	226	22.1	4.51
Q3ZB98	*Bcas1*	Breast carcinoma-amplified sequence 1 homolog (Fragment)	r	3.2	1	1	1	1	555	58.6	5.73
P70562	*Bhlha15*	Class A basic helix-loop-helix protein 15	r	4.6	2	1	1	1	197	22.2	11.15
Q62717	*Cadps*	Calcium-dependent secretion activator 1	r	1.9	2	2	2	4	1289	146.2	5.82
I3MMR5	*Calm3*	Calmodulin 3	St	44.6	6	6	6	73	148	16.7	4.22
P11798	*Camk2a*	Calcium/calmodulin-dependent protein kinase type II subunit alpha	m	18.4	2	4	7	59	478	54.1	7.08

P08413	*Camk2b*	Calcium/calmodulin-dependent protein kinase type II subunit beta	r	35.1	10	7	13	92	542	60.4	7.17
Q8CCM0	*Camk2d*	Calcium/calmodulin-dependent protein kinase type II subunit delta	m	16.9	3	1	5	117	361	40.7	7.78
P11730	*Camk2g*	Calcium/calmodulin-dependent protein kinase type II subunit gamma	r	14.8	2	1	6	46	527	59.0	7.71
P13234	*Camk4*	Calcium/calmodulin-dependent protein kinase type IV	r	3.4	1	1	1	4	474	53.1	4.92
Q63092	*Camkv*	CaM kinase-like vesicle-associated protein	r	12.9	2	5	5	17	504	54.1	5.54
Q80VC9	*Camsap3*	Calmodulin-regulated spectrin-associated protein 3	m	3.8	1	4	4	7	1252	135.1	8.43
P35565	*Canx*	Calnexin	r	2.0	1	1	1	1	591	67.2	4.63
B2GUZ5	*Capza1*	F-actin-capping protein subunit alpha-1	r	14.3	3	2	3	24	286	32.9	5.69
P47754	*Capza2*	F-actin-capping protein subunit alpha-2	m	20.6	1	3	4	31	286	32.9	5.85
A2AMW0	*Capzb*	Capping protein (Actin filament) muscle Z-line, beta	m	48.5	4	1	13	71	260	29.3	6.92
P47757	*Capzb*	F-actin-capping protein subunit beta	m	45.9	2	1	13	70	277	31.3	5.74
D3ZBX9	*Ccdc92*	Coiled-coil domain-containing 92	r	5.1	1	1	1	2	314	35.1	9.38
Q5XIM9	*Cct2*	T-complex protein 1 subunit beta	r	3.7	3	2	2	4	535	57.4	6.46
Q6P502	*Cct3*	T-complex protein 1 subunit gamma	r	19.8	3	10	10	17	545	60.6	6.64
P80315	*Cct4*	T-complex protein 1 subunit delta	m	11.1	2	5	5	14	539	58.0	8.02
Q68FQ0	*Cct5*	T-complex protein 1 subunit epsilon	r	7.6	2	4	4	7	541	59.5	5.72
P80317	*Cct6a*	T-complex protein 1 subunit zeta	m	5.5	4	3	3	5	531	58.0	7.08
D4AC23	*Cct7*	T-complex protein 1 subunit eta	r	6.8	2	3	3	8	544	59.6	8.00
D4ACB8	*Cct8*	Chaperonin subunit 8 (Theta) (Predicted), isoform CRA_a	r	17.2	2	8	8	20	548	59.6	5.54
P60766	*Cdc42*	Cell division control protein 42 homolog	m	11.0	6	1	2	4	191	21.2	6.55
B2RQQ7	*Cdc42bpb*	CDC42 binding protein kinase beta	m	3.0	6	3	4	6	1713	194.6	6.46
Q6A065	*Cep170*	Centrosomal protein of 170 kDa	m	6.1	1	7	7	9	1588	174.9	7.17
P18760	*Cfl1*	Cofilin-1	m	15.1	3	3	3	21	166	18.5	8.09
B7ZMS6	*Chtop*	Chromatin target of PRMT1	m	7.3	2	1	1	3	178	19.4	12.00
P60826	*Cirbp*	Cold-inducible RNA-binding protein	Cg	6.4	1	1	1	5	172	18.6	9.61
A2AGT5	*Ckap5*	Cytoskeleton-associated protein 5	m	2.4	1	3	4	7	2032	225.5	7.96
P07335	*Ckb*	Creatine kinase B-type	r	21.8	2	5	6	18	381	42.7	5.67
Q99JD4	*Clasp2*	CLIP-associating protein 2	r	15.6	4	15	16	30	1286	140.6	8.57
Q922J3	*Clip1*	CAP-Gly domain-containing linker protein 1	m	11.5	1	7	14	31	1391	155.7	5.24
G3V949	*Clip2*	CAP-Gly domain-containing linker protein 2	r	20.9	3	15	19	51	1047	115.7	6.54
B1AWE0	*Clta*	Clathrin light chain A	m	7.9	8	2	2	5	216	23.5	4.50

Q3TWZ9	*Cltb*	Clathrin light chain	m	8.5	3	2	2	5	211	23.2	4.68
P11442	*Cltc*	Clathrin heavy chain 1	r	10.3	3	15	15	41	1675	191.5	5.72
Q4KM73	*Cmpk1*	UMP-CMP kinase	r	6.1	2	1	1	1	196	22.2	5.83
P37397	*Cnn3*	Calponin-3	r	11.2	2	3	3	11	330	36.4	5.72
Q5M7A7	*Cnrip1*	CB1 cannabinoid receptor-interacting protein 1	r	9.8	3	1	1	2	164	18.6	7.85
P12960	*Cntn1*	Contactin-1	m	2.9	2	3	3	5	1020	113.3	6.16
P23514	*Copb1*	Coatomer subunit beta	r	1.5	2	1	1	2	953	106.9	5.96
O35142	*Copb2*	Coatomer subunit beta'	r	1.9	2	1	1	1	905	102.5	5.27
Q91ZN1	*Coro1a*	Coronin-1A	r	19.5	3	8	8	12	461	51.0	6.48
G3V940	*Coro1b*	Coronin	r	4.6	3	2	2	2	484	53.9	5.77
G3V624	*Coro1c*	Coronin	r	16.7	5	8	9	50	474	53.1	7.08
Q8BH44	*Coro2b*	Coronin-2B	m	12.1	1	5	5	8	480	54.9	8.27
P11240	*Cox5a*	Cytochrome c oxidase subunit 5A, mitochondrial	r	10.3	2	1	1	1	146	16.1	6.54
Q5PR69	*Crad*	Capping protein inhibiting regulator of actin dynamics	m	2.0	2	2	2	4	1207	132.2	5.40
P97427	*Crmp1*	Dihydropyrimidinase-related protein 1	m	14.5	3	5	7	38	572	62.1	7.12
Q9CZU6	*Cs*	Citrate synthase, mitochondrial	m	12.5	7	5	5	9	464	51.7	8.57
D3ZPR0	*Cse1l*	Chromosome segregation 1-like (S. cerevisiae) (Predicted)	r	0.8	2	1	1	9	971	110.1	5.77
Q8BK63	*Csnk1a1*	Casein kinase I isoform alpha	m	9.8	3	2	2	7	337	38.9	9.57
Q9JJ76	*Csnk1e*	Casein kinase 1 epsilon	r	3.1	3	1	1	1	416	47.3	9.66
Q9D0E8	*Csnk2a1*	Casein kinase II, alpha 1 polypeptide, isoform CRA_b	m	5.7	3	1	1	1	248	29.5	7.94
P67871	*Csnk2b*	Casein kinase II subunit beta	m	15.8	2	3	3	3	215	24.9	5.55
P47875	*Csrp1*	Cysteine and glycine-rich protein 1	r	8.8	2	1	1	3	193	20.6	8.57
P26231	*Ctnna1*	Catenin alpha-1	m	2.7	2	1	2	5	906	100.0	6.23
Q61301	*Ctnna2*	Catenin alpha-2	m	6.5	2	3	5	11	953	105.2	5.71
Q02248	*Ctnnb1*	Catenin beta-1	m	11.1	2	6	7	16	781	85.4	5.86
O35927	*Ctnnd2*	Catenin delta-2	m	3.8	1	3	3	5	1247	134.9	7.65
Q66HL2	*Cttn*	Cortactin	r	15.9	4	6	6	19	509	56.9	5.24
Q5SQX6	*Cyfip2*	Cytoplasmic FMR1-interacting protein 2	m	4.2	1	2	5	7	1253	145.6	7.05
Q07266	*Dbn1*	Drebrin	r	37.8	3	25	25	169	707	77.4	4.50
Q9JHL4	*Dbnl*	Drebrin-like protein	r	3.2	1	1	1	3	436	48.6	4.92
Q9JLM8	*Dclk1*	Serine/threonine-protein kinase DCLK1	m	23.4	6	14	15	70	756	84.1	8.87
Q5MPA9	*Dclk2*	Serine/threonine-protein kinase DCLK2	r	15.4	2	10	10	19	767	84.0	8.73
G3V7A8	*Dctn1*	Dynactin 1, isoform CRA_a	r	11.9	3	12	12	22	1281	141.9	5.90
Q6AYH5	*Dctn2*	Dynactin subunit 2	r	24.9	2	7	7	27	402	44.1	5.26

D4A1B8	*Dctn3*	Dynactin subunit 3	r	4.3	1	1	1	1	186	21.1	5.71
D3Z6H3	*Dctn6*	Dynactin 6, isoform CRA_b	m	5.4	3	1	1	1	185	20.0	6.38
Q3V349	*Dcx*	Doublecortin, isoform CRA_b	m	24.8	7	6	7	26	331	37.1	9.00
A2ADY9	*Ddi2*	Protein DDI1 homolog 2	m	13.0	1	4	4	14	399	44.6	5.05
I0J0A0	*Ddx3y*	DEAD (Asp-Glu-Ala-Asp) box polypeptide 3, Y-linked (Fragment)	Tm	5.2	8	3	3	4	559	62.8	7.30
Q61656	*Ddx5*	Probable ATP-dependent RNA helicase DDX5	m	10.3	8	5	6	14	614	69.2	8.92
P08461	*Dlat*	Dihydrolipoyllysine-residue acetyltransferase component of pyruvate dehydrogenase complex, mitochondrial]	r	8.2	3	4	4	4	632	67.1	8.53
O35824	*Dnaja2*	DnaJ homolog subfamily A member 2	r	5.6	2	2	2	6	412	45.7	6.48
O08553	*Dpysl2*	Dihydropyrimidinase-related protein 2	m	23.1	1	8	10	68	572	62.2	6.38
Q62952	*Dpysl3*	Dihydropyrimidinase-related protein 3	r	32.6	3	11	14	110	570	61.9	6.49
Q9EQF6	*Dpysl5*	Dihydropyrimidinase-related protein 5	m	7.8	2	4	4	11	564	61.5	7.09
Q7M0E3	*Dstn*	Destrin	r	14.6	2	2	2	2	165	18.5	8.03
P38650	*Dync1h1*	Cytoplasmic dynein 1 heavy chain 1	r	16.3	1	2	65	193	4644	531.9	6.46
Q9JHU4	*Dync1h1*	Cytoplasmic dynein 1 heavy chain 1	m	16.1	1	2	65	182	4644	531.7	6.42
G3V792	*Dync1i1*	Cytoplasmic dynein 1 intermediate chain 1	r	4.0	2	2	2	3	643	72.6	5.12
A2BFF8	*Dync1i2*	Cytoplasmic dynein 1 intermediate chain 2	m	5.2	11	2	2	2	611	68.2	5.29
G3V7G0	*Dync1li1*	Cytoplasmic dynein 1 light intermediate chain 1	r	18.6	3	7	7	12	523	56.6	6.29
Q5D023	*Dync1li2*	Cytoplasmic dynein 1 light intermediate chain 2	r	12.4	3	5	5	14	492	54.1	6.16
P63168	*Dynll1*	Dynein light chain 1, cytoplasmic	m	37.1	2	1	2	10	89	10.4	7.40
Q9D0M5	*Dynll2*	Dynein light chain 2, cytoplasmic	m	44.9	2	2	3	12	89	10.3	7.37
P62627	*Dynlrb1*	Dynein light chain roadblock-type 1	m	12.5	2	1	1	7	96	11.0	7.25
P51807	*Dynlt1*	Dynein light chain Tctex-type 1	m	14.2	2	1	1	4	113	12.5	5.08
G3V771	*Edn2*	Endothelin 2	r	6.3	2	1	1	1	176	19.5	9.86
P62629	*Eef1a1*	Elongation factor 1-alpha 1	Cg	14.9	2	7	7	49	462	50.1	9.01
B5DEN5	*Eef1b2*	Eukaryotic translation elongation factor 1 beta 2	r	12.4	3	2	2	2	225	24.7	4.72
G3V732	*Eef1d*	Elongation factor 1-delta	r	4.7	3	1	1	4	257	28.7	4.97
Q68FR6	*Eef1g*	Elongation factor 1-gamma	r	3.0	1	1	1	2	437	50.0	6.74
P05197	*Eef2*	Elongation factor 2[EF2_RAT]	r	4.2	4	3	3	3	858	95.2	6.83
Q6ZWX6	*Eif2s1*	Eukaryotic translation initiation factor 2 subunit 1	m	6.4	1	2	2	4	315	36.1	5.08
P60843	*Eif4a1*	Eukaryotic initiation factor 4A-I	m	9.4	3	3	3	4	406	46.1	5.48
P70372	*Elavl1*	ELAV-like protein 1	m	6.4	1	2	2	3	326	36.1	9.04
Q60900	*Elavl3*	ELAV-like protein 3	m	12.5	9	3	4	7	367	39.5	9.28

P04764	*Eno1*	Alpha-enolase	r	13.4	11	4	5	8	434	47.1	6.57
D4A361	*Epb4.1l2*	Erythrocyte membrane protein band 4.1-like 2	r	1.5	3	1	1	1	823	91.1	5.49
Q9WTP0	*Epb4111*	Neuronal protein 4.1	r	4.6	7	3	3	6	879	98.2	5.69
Q6TXE9	*Eprs*	Glutamyl-prolyl-tRNA synthetase	r	2.2	2	2	2	5	1486	166.7	7.50
P84089	*Erh*	Enhancer of rudimentary homolog	m	26.9	2	2	2	6	104	12.3	5.92
O08719	*Evl*	Ena/VASP-like protein	r	13.5	2	3	3	6	393	42.1	8.65
Q5SUT0	*Ewsr1*	Ewing sarcoma breakpoint region 1	m	5.3	6	2	2	3	618	64.9	9.38
P55053	*Fabp5*	Fatty acid-binding protein, epidermal	r	30.4	1	3	3	9	135	15.0	7.18
P51880	*Fabp7*	Fatty acid-binding protein, brain	m	22.7	2	2	2	6	132	14.9	5.63
B0BMZ1	*Fam241b*	Family with sequence similarity 241 member B	r	15.8	1	1	1	3	120	13.2	11.41
Q3TJZ6	*Fam98a*	Family with sequence similarity 98, member A protein	m	2.7	2	1	1	1	515	55.0	8.95
P12785	*Fasn*	Fatty acid synthase	r	3.4	2	7	7	11	2505	272.5	6.39
G3V6L9	*Fkbp3*	Peptidyl-prolyl cis-trans isomerase	r	9.8	2	2	2	5	224	25.2	9.28
Q5RKI5	*Flii*	Flightless I homolog (Drosophila)	r	6.7	2	8	8	18	1270	144.8	6.00
B7FAV1	*Flna*	Filamin, alpha (Fragment)	m	0.8	5	1	2	2	2583	274.5	5.97
P85845	*Fscn1*	Fascin	r	25.2	1	1	10	76	493	54.5	6.74
Q61553	*Fscn1*	Fascin	m	25.2	1	1	10	74	493	54.5	6.89
Q8CFQ9	*Fus*	Fusion, derived from t(12;16) malignant liposarcoma (Human)	m	14.5	6	4	6	11	517	52.6	9.36
P97855	*G3bp1*	Ras GTPase-activating protein-binding protein 1	m	3.9	1	1	1	1	465	51.8	5.59
Q542W3	*G3bp2*	GTPase activating protein (SH3 domain) binding protein 2	m	11.1	4	3	3	11	449	50.8	5.43
P60521	*Gabarapl2*	Gamma-aminobutyric acid receptor-associated protein-like 2	m	29.1	1	3	3	8	117	13.7	8.10
P23576	*Gabra2*	Gamma-aminobutyric acid receptor subunit alpha-2	r	4.2	2	1	1	4	451	51.1	9.14
P07936	*Gap43*	Neuromodulin	r	15.0	2	2	2	6	226	23.6	4.70
P04797	*Gapdh*	Glyceraldehyde-3-phosphate dehydrogenase	r	29.7	9	2	7	37	333	35.8	8.03
Q64467	*Gapdhs*	Glyceraldehyde-3-phosphate dehydrogenase, testis-specific	m	7.1	4	1	2	24	440	47.6	7.88
B7FAU8	*Gdi1*	Guanosine diphosphate (GDP) dissociation inhibitor 1 (Fragment)	m	20.8	4	2	2	2	149	16.9	4.83
P47819	*Gfap*	Glial fibrillary acidic protein	r	48.8	1	8	23	323	430	49.9	5.44
Q9Z254	*Gipc1*	PDZ domain-containing protein GIPC1	r	21.0	2	5	5	16	333	36.1	5.91
P08050	*Gja1*	Gap junction alpha-1 protein	r	22.5	3	5	5	29	382	43.0	8.76
P13264	*Gls*	Glutaminase kidney isoform, mitochondrial	r	17.8	2	8	8	21	674	74.0	7.87
P10860	*Glud1*	Glutamate dehydrogenase 1, mitochondrial	r	21.2	3	11	11	33	558	61.4	8.00
P09606	*Glul*	Glutamine synthetase	r	3.0	1	1	1	1	373	42.2	7.08

B9EJU1	*Gm1141*	Gene model 1141, (NCBI)	m	2.7	1	1	1	1	475	52.2	4.82
G3XA41	*Gm6055*	Microtubule-associated proteins 1A/1B light chain 3B	m	22.4	4	1	3	20	125	14.6	7.50
G3V6Q6	*Gna11*	Guanine nucleotide binding protein, alpha 11	r	9.8	3	1	3	5	359	42.0	6.29
P04897	*Gnai2*	Guanine nucleotide-binding protein G(i) subunit alpha-2	r	9.9	31	2	3	17	355	40.5	5.45
P59216	*Gnao1*	Guanine nucleotide-binding protein G(o) subunit alpha	Clo	28.8	27	8	9	30	354	40.0	5.53
Q45QM4	*Gnaq*	Guanine nucleotide binding protein alpha q subunit (Fragment)	r	16.0	7	4	4	6	325	38.3	5.67
P16052	*Gnas*	Guanine nucleotide-binding protein G(s) subunit alpha	Clo	6.9	25	1	2	16	394	45.6	5.82
P62874	*Gnb1*	Guanine nucleotide-binding protein G(I)/G(S)/G(T) subunit beta-1	m	13.5	7	2	4	23	340	37.4	6.00
D3YZX3	*Gnb2*	Guanine nucleotide-binding protein G(I)/G(S)/G(T) subunit beta-2	m	15.5	11	2	4	22	296	32.4	6.15
P68040	*Gnb2l1*	Guanine nucleotide-binding protein subunit beta-2-like 1	m	10.7	1	3	3	16	317	35.1	7.69
Q45QK6	*Gng2*	Guanine nucleotide-binding protein subunit gamma (Fragment)	r	58.8	2	2	2	4	51	5.7	5.94
P35802	*Gpm6a*	Neuronal membrane glycoprotein M6-a	m	5.0	2	1	1	1	278	31.1	5.27
O35127	*Grcc10*	Gene rich cluster C10 protein	m	22.2	1	2	2	2	126	13.2	5.14
Q9JHZ4	*Gripap1*	GRIP1-associated protein 1	r	1.2	1	1	1	1	837	96.0	5.21
P23785	*Grn*	Granulins	r	2.7	4	1	1	2	588	63.3	6.47
Q68FP1	*Gsn*	Gelsolin	r	12.1	5	7	7	28	780	86.0	6.09
G5ALS1	*Gw7_03778*	Keratin, type II cytoskeletal 6B	Hg	10.3	6	2	6	22	565	60.3	7.94
Q02874	*H2afy*	Core histone macro-H2A.1	r	2.4	3	1	1	1	371	39.5	9.79
P0C0S6	*H2afz*	Histone H2A.Z	m	20.3	27	3	3	64	128	13.5	10.58
Q00715	*H2B1*	Histone H2B type 1	r	27.2	22	4	4	12	125	14.0	10.36
P02301	*H3f3c*	Histone H3.3C	m	11.8	7	2	2	3	136	15.3	11.14
Q923W4	*Hdgfrp3*	Hepatoma-derived growth factor-related protein 3	r	15.4	2	2	2	2	202	22.4	8.40
B0R1E3	*Hint1*	Histidine triad nucleotide binding protein	m	11.8	2	1	1	5	119	13.4	5.05
P43275	*Hist1h1a*	Histone H1.1	m	5.2	2	1	1	1	213	21.8	10.93
D3ZBN0	*Hist1h1b*	Histone H1.5	r	14.4	2	3	3	20	222	22.6	10.96
P15864	*Hist1h1c*	Histone H1.2	m	11.8	7	4	4	30	212	21.3	11.00
P62806	*Hist1h4a*	Histone H4	m	47.6	1	5	5	35	103	11.4	11.36
Q8K585	*Hmga1*	High mobility group protein HMG-I/HMG-Y	r	23.4	4	3	3	5	107	11.7	10.32
O88791	*Hmga2*	High mobility group AT-hook 2	r	12.2	1	1	1	1	107	11.7	10.59
P18608	*Hmgn1*	Non-histone chromosomal protein HMG-14	m	15.6	2	1	1	1	96	10.1	9.76

P09602	*Hmgn2*	Non-histone chromosomal protein HMG-17	m	16.7	5	1	1	3	90	9.4	9.99
Q66H40	*Hmgn3*	High mobility group nucleosome-binding domain-containing protein 3	r	15.8	1	1	1	2	95	10.2	10.10
Q9CX86	*Hnrnpa0*	Heterogeneous nuclear ribonucleoprotein A0	m	11.5	1	2	2	2	305	30.5	9.31
P04256	*Hnrnpa1*	Heterogeneous nuclear ribonucleoprotein A1	r	20.3	5	6	6	22	320	34.2	9.14
A7VJC2	*Hnrnpa2b1*	Heterogeneous nuclear ribonucleoproteins A2/B1	r	33.1	2	8	9	29	353	37.5	8.95
A2AL12	*Hnrnpa3*	Heterogeneous nuclear ribonucleoprotein A3	m	19.5	5	5	5	13	318	34.5	9.10
Q9D6G1	*Hnrnpab*	Heterogeneous nuclear ribonucleoprotein A/B, isoform CRA_b	m	5.1	6	1	1	2	276	29.9	6.42
Q9Z204	*Hnrnpc*	Heterogeneous nuclear ribonucleoproteins C1/C2	m	4.2	1	1	1	2	313	34.4	5.05
O35737	*Hnrnph1*	Heterogeneous nuclear ribonucleoprotein H	m	14.9	8	5	5	15	449	49.2	6.30
B2M1R6	*Hnrnpk*	Heterogeneous nuclear ribonucleoprotein K	m	19.6	4	8	8	19	440	48.5	5.54
Q8R081	*Hnrnpl*	Heterogeneous nuclear ribonucleoprotein L	m	5.5	1	1	1	1	586	63.9	8.10
Q3THB3	*Hnrnpm*	Heterogeneous nuclear ribonucleoprotein M	m	10.4	5	6	6	10	690	73.7	8.75
Q566E4	*Hnrnpr*	Heterogeneous nuclear ribonucleoprotein R	r	6.5	5	4	4	5	632	70.8	8.13
G3XA10	*Hnrnpu*	Heterogeneous nuclear ribonucleoprotein U	m	2.8	3	2	2	8	793	86.8	5.92
G3V6A4	*Hnrpd*	Heterogeneous nuclear ribonucleoprotein D, isoform CRA_b	r	13.8	8	3	3	13	304	32.7	8.16
Q921F4	*Hnrpll*	Heterogeneous nuclear ribonucleoprotein L-like	m	2.5	1	1	1	4	591	64.1	5.85
Q9Z214	*Homer1*	Homer protein homolog 1	r	12.6	6	4	4	9	366	41.3	5.53
Q8BUK6	*Hook3*	Protein Hook homolog 3	m	6.0	2	4	4	4	718	83.2	5.19
Q3TKB9	*Hsp90aa1*	heat shock protein 90, alpha (cytosolic), class A member 1 (Fragment)	m	14.9	10	3	7	28	556	64.7	4.98
P11499	*Hsp90ab1*	Heat shock protein HSP 90-beta	m	15.8	3	4	9	30	724	83.2	5.03
P06761	*Hspa5*	Heat shock protein 70 family protein 5	r	23.4	5	10	12	41	654	72.3	5.16
P19378	*HSPA8*	Heat shock cognate 71 kDa protein	Cg	36.7	13	17	19	125	646	70.8	5.36
P48721	*Hspa9*	Stress-70 protein, mitochondrial	r	14.4	4	8	8	12	679	73.8	6.30
P18687	*Hspd1*	60 kDa heat shock protein, mitochondrial	Cg	5.8	2	3	3	6	573	61.0	6.04
Q66HA8	*Hsph1*	Heat shock protein 105 kDa	r	3.4	5	1	2	2	858	96.4	5.55
O88844	*Idh1*	Isocitrate dehydrogenase [NADP] cytoplasmic	m	8.2	4	3	3	3	414	46.6	7.17
Q9CXY6	*Ilf2*	Interleukin enhancer-binding factor 2	m	10.3	2	3	3	3	390	43.0	5.26
P23565	*Ina*	Alpha-internexin	r	34.7	1	2	19	135	505	56.1	5.22
G3V8Q2	*Ina*	Alpha-internexin	r	34.2	2	1	19	126	506	56.2	5.20
Q5SYL3	*Kiaa0100*	Protein KIAA0100	m	0.4	1	1	1	1	2234	254.3	7.34
Q9QXL1	*Kif21b*	Kinesin-like protein KIF21B	m	11.4	1	14	15	31	1668	186.1	7.08

P28740	*Kif2a*	Kinesin-like protein KIF2A	m	9.8	7	6	7	12	705	79.7	6.73
G3V6L4	*Kif5c*	Kinesin family member 5C (Predicted)	r	13.7	7	5	10	20	955	109.1	6.19
P52296	*Kpnb1*	Importin subunit beta-1	r	3.1	2	2	2	4	875	97.1	4.77
Q6IMF3	*Krt1*	Keratin, type II cytoskeletal 1	r	9.1	4	6	6	63	625	64.8	7.87
Q6IFW6	*Krt10*	Keratin, type I cytoskeletal 10	r	11.6	6	3	7	46	526	56.5	5.15
A2A513	*Krt10*	Keratin 10	m	9.6	7	1	5	41	561	57.0	5.07
P08730	*Krt13*	Keratin, type I cytoskeletal 13	m	8.0	17	0	5	24	437	47.7	4.86
Q61781	*Krt14*	Keratin, type I cytoskeletal 14	m	12.0	27	2	6	29	484	52.8	5.17
Q61414	*Krt15*	Keratin, type I cytoskeletal 15	m	9.1	33	0	5	26	452	49.1	4.86
Q6IG02	*Krt2*	Keratin, type II cytoskeletal 2 epidermal	r	5.1	1	2	4	13	685	69.1	7.69
Q61897	*Krt33b*	Keratin, type I cuticular Ha3-II	m	4.0	17	1	2	8	404	45.8	4.82
Q6IG00	*Krt4*	Keratin, type II cytoskeletal 4	r	6.9	3	1	4	22	536	57.6	7.64
Q6P6Q2	*Krt5*	Keratin, type II cytoskeletal 5	r	14.2	2	4	8	38	576	61.8	7.80
Q4FZU2	*Krt6a*	Keratin, type II cytoskeletal 6A	r	7.3	1	1	4	22	552	59.2	7.94
Q3UV17	*Krt76*	Keratin, type II cytoskeletal 2 oral	m	4.9	2	1	3	13	594	62.8	8.43
Q0VDM9	*Krt78*	Krt78 protein (Fragment)	m	4.9	1	1	2	12	492	54.7	6.19
Q8VED5	*Krt79*	Keratin, type II cytoskeletal 79	m	4.5	1	1	2	7	531	57.5	7.69
Q10758	*Krt8*	Keratin, type II cytoskeletal 8	r	3.7	4	1	2	5	483	54.0	6.00
P04642	*Ldha*	L-lactate dehydrogenase A chain	r	6.6	8	2	2	4	332	36.4	8.27
G3V8I6	*Lima1*	Epithelial protein lost in neoplasm	r	17.4	3	9	9	27	755	83.7	6.25
Q3UH68	*Limch1*	LIM and calponin homology domains-containing protein 1	m	11.0	1	9	9	13	1057	118.1	5.48
G3V7U4	*Lmnb1*	Lamin-B1	r	12.6	3	6	6	7	587	66.6	5.16
G3V957	*Loc100359960*	S5 DRBM domain-containing protein	r	16.5	3	4	4	6	267	28.6	9.76
D3ZHA7	*Loc684533*	Similar to myosin light chain 1 slow a	r	33.3	2	5	6	21	207	22.8	5.71
G5E8E1	*Lrrfip1*	Leucine rich repeat (In FLII) interacting protein 1, isoform CRA_e	m	8.4	3	1	3	12	428	48.9	5.57
A6H5U5	*Lrrfip1*	Lrrfip1 protein	m	5.3	3	1	3	17	663	75.1	5.85
Q4V7E8	*Lrrfip2*	Leucine-rich repeat flightless-interacting protein 2	r	14.4	2	4	5	18	437	49.7	5.95
D3ZHV2	*Macf1*	Microtubule-actin cross-linking factor 1	r	10.4	5	49	49	112	5430	619.2	5.40
P34926	*Map1a*	Microtubule-associated protein 1A	r	1.7	4	3	4	23	2774	299.3	4.94
P15205	*Map1b*	Microtubule-associated protein 1B	r	9.0	3	8	18	72	2459	269.3	4.81
Q91VR7	*Map1lc3a*	Microtubule-associated proteins 1A/1B light chain 3A	m	23.1	1	1	3	30	121	14.3	8.68
P0C5W1	*Map1s*	Microtubule-associated protein 1S	r	1.3	1	1	1	1	972	102.7	7.11
P15146	*Map2*	Microtubule-associated protein 2	r	11.6	10	15	15	56	1861	202.3	4.86

Q5M7W5	*Map4*	Microtubule-associated protein 4	r	12.3	2	6	9	14	1057	110.2	4.73
P27546	*Map4*	Microtubule-associated protein 4	m	4.3	2	1	4	6	1125	117.4	4.98
P97820	*Map4k4*	Mitogen-activated protein kinase kinase kinase kinase 4	m	2.2	12	2	2	3	1233	140.5	7.47
Q63560	*Map6*	Microtubule-associated protein 6	r	11.9	2	7	7	15	952	100.4	9.44
P63085	*Mapk1*	Mitogen-activated protein kinase 1	m	4.2	3	1	1	1	358	41.2	6.98
Q9R237	*Mapk8ip1*	C-Jun-amino-terminal kinase-interacting protein 1	r	5.9	3	3	3	4	708	77.3	4.96
Q61166	*Mapre1*	Microtubule-associated protein RP/EB family member 1	m	7.5	2	1	1	1	268	30.0	5.22
Q3B8Q0	*Mapre2*	Microtubule-associated protein RP/EB family member 2	r	8.3	3	2	2	2	326	37.0	5.38
A0JN25	*Mapt*	Microtubule-associated protein	r	34.7	10	8	8	16	343	35.9	9.44
Q547J4	*Mapt*	Microtubule-associated protein	m	21.8	9	1	6	23	372	38.9	9.50
P30009	*Marcks*	Myristoylated alanine-rich C-kinase substrate	r	25.2	2	6	6	24	309	29.8	4.34
Q9EPH2	*Marcksl1*	MARCKS-related protein	r	14.1	2	2	2	2	199	19.8	4.65
Q8VHF0	*Mark3*	MAP/microtubule affinity-regulating kinase 3	r	6.9	2	2	4	6	797	88.7	9.41
Q8CIP4	*Mark4*	MAP/microtubule affinity-regulating kinase 4	m	2.4	2	1	2	3	752	82.6	9.67
P04636	*Mdh2*	Malate dehydrogenase, mitochondrial	r	20.4	4	6	6	12	338	35.7	8.68
Q9Z2D6	*Mecp2*	Methyl-CpG-binding protein 2	m	3.9	2	1	1	1	484	52.3	9.96
G3V9F3	*Mprip*	Myosin phosphatase Rho-interacting protein	r	5.8	4	1	5	13	1029	116.9	6.21
B2RQQ5	*Mtap1b*	Microtubule-associated protein 1B	m	5.6	4	2	12	60	2464	270.1	4.83
O35821	*Mybbp1a*	Myb-binding protein 1A	r	0.6	1	1	1	1	1344	152.2	8.95
Q7TPH6	*Mycbp2*	Probable E3 ubiquitin-protein ligase MYCBP2	m	0.7	2	3	3	3	4711	517.4	7.12
A7UQY4	*Myef2*	Myelin basic protein expression factor 2, repressor	m	6.4	4	3	3	5	530	57.1	9.16
Q5SV64	*Myh10*	Myosin, heavy polypeptide 10, non-muscle	m	52.2	17	4	108	1535	2007	232.3	5.54
Q9JLT0	*Myh10*	Myosin-10	r	42.0	22	1	87	696	1976	228.8	5.60
B1AR69	*Myh13*	Myosin, heavy polypeptide 13, skeletal muscle	m	0.9	12	1	2	16	1938	223.4	5.57
Q6URW6	*Myh14*	Myosin-14	m	13.2	22	14	21	82	2000	228.4	5.55
G3V6P7	*Myh9*	Myosin, heavy polypeptide 9, non-muscle	r	31.5	24	2	53	228	1960	226.3	5.66
Q60605	*Myl6*	Myosin light polypeptide 6	m	47.7	11	7	8	127	151	16.9	4.65
B2RRE2	*Myo18a*	Unconventional myosin-XVIIIa	m	15.9	2	28	28	86	2047	232.0	6.10
Q05096	*Myo1b*	Unconventional myosin-Ib	r	7.2	3	7	7	8	1136	131.8	9.36
Q63355	*Myo1c*	Unconventional myosin-Ic	r	4.1	2	4	4	5	1044	119.7	9.39
Q9QYF3	*Myo5a*	Unconventional myosin-Va	r	31.7	5	7	56	359	1828	211.6	8.69
Q99104	*Myo5a*	Unconventional myosin-Va	m	29.9	6	4	53	348	1853	215.4	8.63
P70569	*Myo5b*	Unconventional myosin-Vb	r	5.2	4	1	10	59	1846	213.6	6.98
B2RWR8	*Myo6*	Myosin VI	m	13.5	2	15	15	84	1262	145.8	8.47

Q60817	*Naca*	Nascent polypeptide-associated complex subunit alpha	m	19.5	3	3	3	11	215	23.4	4.56
Q3TF41	*Nap1l1*	Nucleosome assembly protein 1-like 1, isoform CRA_d	m	7.3	5	1	2	22	368	42.7	4.55
Q5U2Z3	*Nap1l4*	Nucleosome assembly protein 1-like 4	r	12.7	8	4	4	20	386	43.9	4.68
P85969	*Napb*	Beta-soluble NSF attachment protein	r	13.8	2	3	3	3	297	33.4	5.47
Q9EPN1	*Nbea*	Neurobeachin	m	3.8	2	9	9	11	2936	326.5	6.20
P13596	*Ncam1*	Neural cell adhesion molecule 1	r	2.9	2	2	2	2	858	94.6	4.92
O35095	*Ncdn*	Neurochondrin	r	4.3	2	3	3	5	729	78.9	5.62
P28660	*Nckap1*	Nck-associated protein 1	m	3.6	3	4	4	6	1128	128.7	6.62
P13383	*Ncl*	Nucleolin	r	4.9	5	3	3	4	713	77.1	4.74
Q62425	*Ndufa4*	NADH dehydrogenase [ubiquinone] 1 alpha subcomplex subunit 4	m	24.4	1	2	2	2	82	9.3	9.52
Q5RJN0	*Ndufs7*	NADH dehydrogenase (Ubiquinone) Fe-S protein 7	r	9.2	1	1	1	2	218	23.9	9.99
P19527	*Nefl*	Neurofilament light polypeptide	r	27.1	2	12	14	78	542	61.3	4.65
G3V7S2	*Nefm*	Neurofilament 3, medium	r	19.1	3	12	15	69	845	95.7	4.79
G3V8F8	*Nes*	Nestin, isoform CRA_b	r	6.8	4	11	11	18	1893	208.8	4.32
Q5NC80	*Nme1*	Nucleoside diphosphate kinase (Fragment)	m	13.4	5	1	1	2	127	14.1	9.09
Q99K48	*Nono*	Non-POU domain-containing octamer-binding protein	m	4.4	2	2	2	7	473	54.5	8.95
Q5SQB0	*Npm1*	Nucleophosmin	m	13.3	6	3	3	12	264	29.5	4.59
Q9QUL6	*Nsf*	Vesicle-fusing ATPase	r	19.5	3	13	13	45	744	82.6	6.99
Q9CQF3	*Nudt21*	Cleavage and polyadenylation specificity factor subunit 5	m	3.5	1	1	1	1	227	26.2	8.82
Q5XI78	*Ogdh*	2-oxoglutarate dehydrogenase, mitochondrial	r	1.2	2	1	1	2	1023	116.2	6.77
Q5DJU3	*Orc3*	Origin recognition complex subunit 3	Sc	1.1	1	1	1	1	713	82.3	8.06
Q05BN2	*Pa2g4*	Proliferation-associated 2G4 protein (Fragment)	m	14.3	3	5	5	20	372	41.5	7.50
P29341	*Pabpc1*	Polyadenylate-binding protein 1	m	9.0	13	5	5	9	636	70.6	9.50
P63005	*Pafah1b1*	Platelet-activating factor acetylhydrolase IB subunit alpha	m	21.2	2	6	6	22	410	46.6	7.37
P51583	*Paics*	Multifunctional protein ADE2	r	2.6	2	1	1	1	425	47.1	7.69
P60335	*Pcbp1*	Poly(rC)-binding protein 1	m	8.7	1	2	3	7	356	37.5	7.09
Q61990	*Pcbp2*	Poly(rC)-binding protein 2	m	13.3	4	3	4	16	362	38.2	6.79
Q9WUJ3	*Pde4dip*	Myomegalin	r	1.7	4	3	3	3	2324	261.9	5.40
P49432	*Pdhb*	Pyruvate dehydrogenase E1 component subunit beta, mitochondrial	r	9.5	3	3	3	7	359	39.0	6.65
Q7TQ85	*Pdhx*	Pyruvate dehydrogenase complex, component X	r	4.8	5	2	2	5	539	58.8	8.53
P47857	*Pfkm*	6-phosphofructokinase, muscle type	m	2.8	3	2	2	4	780	85.2	8.00
Q5SX49	*Pfn1*	Profilin	m	10.7	2	1	1	2	112	11.8	4.88

G3V9N1	*Pgam5*	Serine/threonine-protein phosphatase PGAM5, mitochondrial	r	11.8	4	3	3	6	288	32.0	8.68
P09411	*Pgk1*	Phosphoglycerate kinase 1	m	2.6	3	1	1	1	417	44.5	7.90
P86220	*PHB*	Prohibitin (Fragments)	Ma	11.5	4	1	1	1	87	10.0	4.60
O35129	*Phb2*	Prohibitin-2	m	4.0	2	1	1	4	299	33.3	9.83
O08651	*Phgdh*	D-3-phosphoglycerate dehydrogenase	r	6.4	2	3	3	4	533	56.5	6.71
Q66WT9	*Picalm*	Clathrin-assembly lymphoid myeloid leukemia protein	r	4.5	4	1	2	2	597	64.7	9.01
O88377	*Pip4k2b*	Phosphatidylinositol 5-phosphate 4-kinase type-2 beta	r	3.4	2	1	1	1	416	47.2	7.52
P11980	*Pkm*	Pyruvate kinase isozymes M1/M2	r	4.5	2	2	2	3	531	57.8	7.06
Q6S3A1	*Plec*	Plectin	r	2.5	11	9	9	13	4451	506.4	5.68
P10111	*Ppia*	Peptidyl-prolyl cis-trans isomerase A	r	20.7	4	3	3	17	164	17.9	8.16
P24368	*Ppib*	Peptidyl-prolyl cis-trans isomerase B	r	17.1	2	3	3	18	216	23.8	9.50
A2BGI8	*Ppih*	Peptidyl-prolyl cis-trans isomerase (Fragment)	m	8.9	2	1	1	1	158	17.2	8.34
P62137	*Ppp1ca*	Serine/threonine-protein phosphatase PP1-alpha catalytic subunit	m	29.7	4	3	9	51	330	37.5	6.33
P62141	*Ppp1cb*	Serine/threonine-protein phosphatase PP1-beta catalytic subunit	m	23.9	2	1	7	37	327	37.2	6.19
P63087	*Ppp1cc*	Serine/threonine-protein phosphatase PP1-gamma catalytic subunit	m	24.2	3	1	7	43	323	37.0	6.54
Q10728	*Ppp1r12a*	Protein phosphatase 1 regulatory subunit 12A	r	6.2	2	5	5	13	1032	115.2	5.47
G3V629	*Ppp1r18*	Protein phosphatase 1, regulatory subunit 18	r	5.9	1	4	4	6	578	63.4	5.52
O35867	*Ppp1r9a*	Neurabin-1	r	10.0	4	8	9	32	1095	122.7	5.14
O35274	*Ppp1r9b*	Neurabin-2	r	23.5	2	13	15	68	817	89.6	4.92
P62715	*Ppp2cb*	Serine/threonine-protein phosphatase 2A catalytic subunit beta isoform	m	7.4	2	1	2	2	309	35.6	5.43
Q76MZ3	*Ppp2r1a*	Serine/threonine-protein phosphatase 2A 65 kDa regulatory subunit A alpha isoform	m	6.8	1	4	4	4	589	65.3	5.11
D3ZHI9	*Ppp2r5e*	Serine/threonine-protein phosphatase 2A 56 kDa regulatory subunit	r	3.0	2	1	1	1	429	50.3	8.16
P63328	*Ppp3ca*	Serine/threonine-protein phosphatase 2B catalytic subunit alpha isoform	m	6.3	3	1	3	4	521	58.6	5.86
G3X8U7	*Ppp3cb*	Serine/threonine-protein phosphatase	m	6.2	6	2	3	3	515	58.1	6.10
Q63810	*Ppp3r1*	Calcineurin subunit B type 1	m	7.1	1	1	1	1	170	19.3	4.81
P35700	*Prdx1*	Peroxiredoxin-1	m	21.6	8	3	3	5	199	22.2	8.12

P35704	*Prdx2*	Peroxiredoxin-2	r	10.6	3	2	2	3	198	21.8	5.59
G3V7I0	*Prdx3*	Peroxiredoxin 3	r	4.7	2	1	1	1	257	28.3	7.55
G3V9U8	*Prkacb*	Protein kinase, cAMP dependent, catalytic, beta (Predicted), isoform CRA_b	r	10.3	7	3	3	3	339	39.5	8.56
P12369	*Prkar2b*	cAMP-dependent protein kinase type II-beta regulatory subunit	r	6.5	3	2	2	5	416	46.1	4.98
G3V7J2	*Prkra*	Interferon-inducible double stranded RNA-dependent protein kinase activator A	r	8.0	3	2	2	3	313	34.4	8.56
Q9D7G0	*Prps1*	Ribose-phosphate pyrophosphokinase 1	m	10.4	3	3	3	6	318	34.8	6.98
P00762	*Prss1*	Anionic trypsin-1	r	8.1	1	1	1	6	246	25.9	4.89
Q566D6	*Psip1*	PC4 and SFRS1-interacting protein	r	10.0	5	2	2	2	331	37.3	9.17
A2AGN7	*Psmc3*	26S protease regulatory subunit 6A	m	3.5	5	1	1	1	400	44.6	5.02
Q8K1K2	*Psmc5*	26S proteasome regulatory subunit 8	m	10.9	2	3	3	3	348	38.7	5.96
Q8CGI9	*Psmd1*	Psmd1 protein (Fragment)	m	7.2	5	5	5	7	824	90.9	6.04
B0BN93	*Psmd13*	26S proteasome non-ATPase regulatory subunit 13	r	16.2	2	6	6	12	376	42.8	5.83
Q4FZT9	*Psmd2*	26S proteasome non-ATPase regulatory subunit 2	r	7.4	4	5	5	7	908	100.1	5.20
P14685	*Psmd3*	26S proteasome non-ATPase regulatory subunit 3	m	14.2	2	7	7	16	530	60.7	8.44
Q6PCT9	*Psmd6*	Proteasome (Prosome, macropain) 26S subunit, non-ATPase, 6	r	6.7	2	2	2	3	389	45.6	5.52
D3ZGY1	*Pym1*	PYM homolog 1, exon junction complex-associated factor	r	5.4	1	1	1	1	203	22.7	9.64
P61027	*Rab10*	Ras-related protein Rab-10	m	22.5	57	3	4	23	200	22.5	8.38
Q8R361	*Rab11fip5*	Rab11 family-interacting protein 5	m	2.8	1	1	1	3	645	69.5	9.07
P63011	*Rab3a*	Ras-related protein Rab-3A	m	8.6	39	1	2	15	220	25.0	5.03
P61294	*Rab6b*	Ras-related protein Rab-6B	m	17.3	34	1	3	16	208	23.4	5.53
O88931	*Rac2*	Ras-related C3 botulinum toxin substrate 2	Cp	10.9	13	1	2	4	192	21.4	7.62
P62827	*Ran*	GTP-binding nuclear protein Ran	m	15.7	5	3	3	3	216	24.4	7.49
Q8R4X3	*Rbm12*	RNA-binding protein 12	m	1.4	2	1	1	7	992	102.7	8.32
Q8C2Q3	*Rbm14*	RNA-binding protein 14	m	1.5	1	1	1	1	669	69.4	9.67
Q91VM5	*Rbmxl1*	RNA binding motif protein, X-linked-like-1	m	18.0	5	1	6	11	388	42.1	9.99
P84586	*Rbmxrtl*	RNA-binding motif protein, X chromosome retrogene-like	r	16.5	7	5	5	16	388	42.2	9.99
Q62703	*Rcn2*	Reticulocalbin-2	r	8.8	2	2	2	5	320	37.4	4.41
Q9QUI0	*Rhoa*	Transforming protein RhoA	m	11.9	5	2	2	4	193	21.8	6.10
P62746	*Rhob*	Rho-related GTP-binding protein RhoB	m	5.6	1	1	1	1	196	22.1	5.24
Q9JIR4	*Rims1*	Regulating synaptic membrane exocytosis protein 1	r	3.9	2	4	4	6	1615	179.5	9.58

P13832	*Rlc-a*	Myosin regulatory light chain RLC-A	r	40.1	5	6	6	107	172	19.9	4.81
Q3TDK6	*Rogdi*	Protein rogdi homolog	m	12.5	2	3	3	4	287	32.1	8.18
I7HLV2	*Rpl10*	60S ribosomal protein L10 (Fragment)	m	12.4	3	2	2	2	201	23.1	10.01
P53026	*Rpl10a*	60S ribosomal protein L10a	m	3.7	2	1	1	2	217	24.9	9.98
D3Z3K1	*Rpl11*	60S ribosomal protein L11	m	12.9	3	2	2	5	178	20.3	9.60
P23358	*Rpl12*	60S ribosomal protein L12	r	40.0	5	4	4	16	165	17.8	9.42
P41123	*Rpl13*	60S ribosomal protein L13	r	24.2	4	5	5	13	211	24.3	11.55
D3YY61	*Rpl13a-ps1*	Ribosomal protein L13a, pseudogene 1 protein	m	5.4	4	1	1	3	203	23.3	10.29
Q63507	*Rpl14*	60S ribosomal protein L14	r	10.3	4	2	2	5	214	23.3	11.11
Q9CZM2	*Rpl15*	60S ribosomal protein L15	m	19.1	2	3	3	6	204	24.1	11.62
P12001	*Rpl18*	60S ribosomal protein L18	r	21.3	3	3	3	10	188	21.6	11.78
P62717	*Rpl18a*	60S ribosomal protein L18a	m	15.3	1	2	2	5	176	20.7	10.71
A2A547	*Rpl19*	Ribosomal protein L19	m	8.8	2	1	1	2	194	23.2	11.47
O09167	*Rpl21*	60S ribosomal protein L21	m	18.1	7	2	2	2	160	18.6	10.49
P67984	*Rpl22*	60S ribosomal protein L22	m	18.8	2	2	2	6	128	14.7	9.19
P62830	*Rpl23*	60S ribosomal protein L23	m	10.7	1	1	1	7	140	14.9	10.51
D3YWP3	*Rpl23a-ps3*	60S ribosomal protein L23a, pseudogene 3 protein	m	20.5	3	3	3	9	156	17.7	10.42
Q8BP67	*Rpl24*	60S ribosomal protein L24	m	19.8	1	3	3	15	157	17.8	11.25
B1ARA3	*Rpl26*	60S ribosomal protein L26 (Fragment)	m	24.3	5	3	3	6	103	12.2	10.92
A2A4Q0	*Rpl27*	60S ribosomal protein L27 (Fragment)	m	10.3	2	1	1	4	87	10.4	10.36
P14115	*Rpl27a*	60S ribosomal protein L27a	m	27.7	2	4	4	9	148	16.6	11.12
P17702	*Rpl28*	60S ribosomal protein L28	r	13.9	2	2	2	11	137	15.8	12.02
P25886	*Rpl29*	60S ribosomal protein L29	r	7.1	1	1	1	1	156	17.3	11.78
P21531	*Rpl3*	60S ribosomal protein L3	r	5.2	3	2	2	7	403	46.1	10.21
P62889	*Rpl30*	60S ribosomal protein L30	m	33.9	1	3	3	4	115	12.8	9.63
Q6QMZ5	*Rpl35*	Ribosomal protein L35 (Fragment)	Cla	9.9	2	1	1	3	101	11.8	11.30
O55142	*Rpl35a*	60S ribosomal protein L35a	m	8.2	1	1	1	2	110	12.5	10.89
D3YW41	*Rpl36-ps3*	60S ribosomal protein L36	m	18.1	4	2	2	4	105	12.2	11.40
P83882	*Rpl36a*	60S ribosomal protein L36a	m	8.5	1	1	1	4	106	12.4	10.58
Q9D8E6	*Rpl4*	60S ribosomal protein L4	m	4.5	3	2	2	2	419	47.1	11.00
P09895	*Rpl5*	60S ribosomal protein L5	r	13.1	3	3	3	5	297	34.4	9.74
P21533	*Rpl6*	60S ribosomal protein L6	r	9.1	3	3	3	5	298	33.5	10.74
B0K031	*Rpl7*	60S ribosomal protein L7	r	3.1	3	1	1	2	260	30.3	10.87
D3YVE6	*Rpl7a-ps10*	Ribosomal protein L7a, pseudogene 10 protein	m	9.0	4	2	2	7	266	30.0	10.45

Q6QMZ3	*Rpl8*	Ribosomal protein L8 (Fragment)	Cla	17.9	2	2	2	2	151	16.3	10.89
G3UW34	*Rpl9-ps6*	Ribosomal protein L9, pseudogene 6 protein	m	11.5	3	2	2	4	192	21.8	9.88
P14869	*Rplp0*	60S acidic ribosomal protein P0	m	17.4	3	4	4	19	317	34.2	6.25
P19944	*Rplp1*	60S acidic ribosomal protein P1	r	29.0	2	2	2	3	114	11.5	4.32
P02401	*Rplp2*	60S acidic ribosomal protein P2	r	50.4	2	3	3	48	115	11.7	4.54
P63325	*Rps10*	40S ribosomal protein S10	m	8.5	1	1	1	3	165	18.9	10.15
P62281	*Rps11*	40S ribosomal protein S11	m	13.3	1	2	2	3	158	18.4	10.30
P63323	*Rps12*	40S ribosomal protein S12	m	20.5	3	2	2	2	132	14.5	7.24
Q921R2	*Rps13*	40S ribosomal protein S13	m	22.1	3	3	3	8	140	16.1	10.71
P13471	*Rps14*	40S ribosomal protein S14	r	15.9	2	2	2	11	151	16.2	10.05
P62842	*Rps15*	40S ribosomal protein S15	Ma	9.0	1	1	1	1	145	17.0	10.39
P14131	*Rps16*	40S ribosomal protein S16	m	26.0	2	4	4	9	146	16.4	10.21
P04644	*Rps17*	40S ribosomal protein S17	r	8.2	2	1	1	4	135	15.5	9.77
P62270	*Rps18*	40S ribosomal protein S18	m	27.0	4	4	4	12	152	17.7	10.99
F6Q5Z8	*Rps19-ps1*	Ribosomal protein S19, pseudogene 1 protein	m	29.0	2	5	5	37	145	16.2	10.52
P60867	*Rps20*	40S ribosomal protein S20	m	19.3	1	2	2	5	119	13.4	9.94
Q8C1L7	*Rps21*	40S ribosomal protein S21	m	12.4	3	1	1	3	81	8.9	8.51
P62298	*RPS23*	40S ribosomal protein S23	Cla	8.4	1	1	1	1	143	15.8	10.49
P62848	*Rps24*	40S ribosomal protein S24	Ma	11.3	2	1	1	1	133	15.4	10.78
P62852	*Rps25*	40S ribosomal protein S25	m	24.0	1	4	4	20	125	13.7	10.11
Q6QMZ1	*Rps26*	40S ribosomal protein S26 (Fragment)	Cla	8.2	3	1	1	1	110	12.3	10.81
Q6ZWU9	*Rps27*	40S ribosomal protein S27	m	25.0	3	2	2	8	84	9.5	9.45
P62983	*Rps27a*	Ubiquitin-40S ribosomal protein S27a	m	42.3	10	1	6	37	156	17.9	9.64
P62858	*Rps28*	40S ribosomal protein S28	m	30.4	2	2	2	10	69	7.8	10.70
P62908	*Rps3*	40S ribosomal protein S3	m	26.3	1	5	5	31	243	26.7	9.66
P49242	*Rps3a*	40S ribosomal protein S3a	r	17.1	4	5	5	11	264	29.9	9.73
P47961	*Rps4*	40S ribosomal protein S4	Cg	24.7	4	5	5	7	263	29.6	10.15
D3YYM6	*Rps5*	40S ribosomal protein S5 (Fragment)	m	13.2	5	2	2	5	182	20.4	9.55
P62754	*Rps6*	40S ribosomal protein S6	m	10.8	1	2	2	5	249	28.7	10.84
P62242	*Rps8*	40S ribosomal protein S8	m	23.1	4	4	4	9	208	24.2	10.32
Q9CXW7	*Rps9*	40S ribosomal protein S9	m	6.5	2	1	1	1	139	16.6	10.80
D3YTT7	*Rpsa-ps10*	40S ribosomal protein SA	m	14.2	4	4	4	15	295	32.8	4.93
P62071	*Rras2*	Ras-related protein R-Ras2	m	5.9	1	1	1	3	204	23.4	6.01
Q99LF4	*Rtcb*	tRNA-splicing ligase RtcB homolog	m	10.3	1	5	5	10	505	55.2	7.23

Q7M6W1	*Rtn1*	Reticulon	m	9.1	3	2	2	3	208	23.5	8.91
D4A1U2	*Rtn3*	Reticulon 3, isoform CRA_a	r	4.3	3	1	1	3	256	27.4	8.24
Q5FVJ0	*Rufy3*	Protein RUFY3	r	17.5	2	7	7	17	469	52.9	5.49
Q9WTM5	*Ruvbl2*	RuvB-like 2	m	10.6	1	4	4	6	463	51.1	5.64
Q6ZPE2	*Sbf1*	Myotubularin-related protein 5	m	0.6	2	1	1	2	1867	208.6	7.12
Q8C8N2	*Scai*	Suppressor of cancer cell invasion protein	m	1.8	1	1	1	3	606	70.2	8.60
Q80UF4	*Sdccag8*	Serologically defined colon cancer antigen 8 homolog	m	1.3	1	1	1	1	717	82.9	6.52
D3ZDY1	*Sept3*	Neuronal-specific septin-3	r	3.0	2	1	1	1	337	38.7	6.81
B5DFG5	*Sept6*	Septin 6	r	3.3	1	1	1	1	427	48.7	6.67
Q6AXS5	*Serbp1*	Plasminogen activator inhibitor 1 RNA-binding protein	r	2.7	2	1	1	1	407	44.7	8.54
A2BE92	*Set*	Protein SET (Fragment)	m	13.9	5	2	2	3	151	17.6	5.16
Q8VIJ6	*Sfpq*	Splicing factor, proline- and glutamine-rich	m	9.3	1	6	6	8	699	75.4	9.44
Q8BG73	*Sh3bgrl2*	SH3 domain-binding glutamic acid-rich-like protein 2	m	11.2	1	1	1	4	107	12.2	5.55
P63209	*Skp1*	S-phase kinase-associated protein 1	Cp	20.3	2	3	3	7	163	18.6	4.54
G3V6A2	*Slain2*	SLAIN motif family, member 2	r	2.3	1	1	1	1	607	65.3	9.72
Q8JZR4	*Slc1a7*	Excitatory amino acid transporter 5	m	1.6	1	1	1	1	559	60.1	6.04
P48962	*Slc25a4*	ADP/ATP translocase 1	m	16.4	4	3	5	11	298	32.9	9.72
P51881	*Slc25a5*	ADP/ATP translocase 2	m	12.8	3	2	4	7	298	32.9	9.73
Q9ET64	*Smpd2*	Sphingomyelin phosphodiesterase 2	r	4.0	1	1	1	1	422	47.6	7.08
Q61548	*Snap91*	Clathrin coat assembly protein AP180	m	4.0	4	2	3	5	901	91.8	4.88
B0BN51	*Snrpb*	Small nuclear ribonucleoprotein-associated protein	r	10.0	5	2	2	2	231	23.6	10.90
Q62241	*Snrpc*	U1 small nuclear ribonucleoprotein C	m	7.6	1	1	1	1	159	17.4	9.67
D3Z5N9	*Snrpd2*	Small nuclear ribonucleoprotein Sm D2	m	17.0	2	2	2	4	118	13.5	9.69
P62320	*Snrpd3*	Small nuclear ribonucleoprotein Sm D3	m	7.9	1	1	1	1	126	13.9	10.32
P62305	*Snrpe*	Small nuclear ribonucleoprotein E	m	13.0	1	1	1	1	92	10.8	9.44
Q9D0T1	*Snu13*	NHP2-like protein 1	m	9.4	1	1	1	1	128	14.2	8.46
E1U8D0	*Soga1*	Suppressor of glucose, autophagy-associated protein 1	m	0.6	2	1	1	1	1418	159.1	6.46
Q62417	*Sorbs1*	Sorbin and SH3 domain-containing protein 1	m	4.3	1	4	4	9	1290	143.0	8.25
Q2KN98	*Specc1l*	Cytospin-A	m	5.6	2	5	5	7	1118	124.4	5.76
Q4V8J7	*Spin1*	Spindlin-1	r	5.0	2	1	1	1	262	29.6	6.96
O70559	*Sprr2h*	Small proline-rich protein 2H	m	41.7	7	1	1	1	108	11.7	7.83
P16086	*Sptan1*	Spectrin alpha chain, non-erythrocytic 1	r	45.7	6	7	103	817	2472	284.5	5.33
A3KGU5	*Sptan1*	Spectrin alpha chain, non-erythrocytic 1	m	51.8	5	2	122	1434	2457	282.7	5.33
A3KGU9	*Sptan1*	Spectrin alpha chain, non-erythrocytic 1 (Fragment)	m	45.7	1	1	20	166	477	55.4	5.55

G3V6S0	*Sptbn1*	Spectrin beta chain	r	41.4	9	76	83	488	2363	273.9	5.59
Q62261	*Sptbn1*	Spectrin beta chain, non-erythrocytic 1	m	49.1	7	1	100	786	2363	274.1	5.58
Q9QWN8	*Sptbn2*	Spectrin beta chain, non-erythrocytic 2	r	17.5	4	28	35	119	2388	270.9	5.83
Q9QXY2	*Srcin1*	SRC kinase signaling inhibitor 1	r	7.7	6	8	8	16	1197	129.7	9.36
Q66H19	*Srfbp1*	Serum response factor-binding protein 1	r	3.2	1	1	1	1	442	49.2	9.61
D4A208	*Srgap2*	SLIT-ROBO Rho GTPase-activating protein 2	r	6.8	3	5	6	10	1071	120.8	6.76
A2AD25	*Srpk1*	SRSF protein kinase 1	m	9.6	2	1	1	3	135	16.0	10.56
Q6PDM2	*Srsf1*	Serine/arginine-rich splicing factor 1	m	22.2	1	5	5	7	248	27.7	10.36
Q3U781	*Srsf3*	RRM domain-containing protein	m	9.7	3	1	1	4	124	14.2	10.08
A2A5R8	*Stau1*	Double-stranded RNA-binding protein Staufen homolog 1	m	2.7	7	1	1	2	485	53.7	9.55
P13668	*Stmn1*	Stathmin	r	18.8	4	3	3	8	149	17.3	5.97
D3ZDD7	*Strbp*	Spermatid perinuclear RNA binding protein, isoform CRA_a	r	1.3	5	1	1	1	672	73.7	8.68
O70257	*Stx7*	Syntaxin-7	r	3.8	1	1	1	2	261	29.8	5.43
O08599	*Stxbp1*	Syntaxin-binding protein 1	m	12.1	1	6	6	8	594	67.5	6.96
P11031	*Sub1*	Activated RNA polymerase II transcriptional coactivator p15	m	15.8	2	2	2	4	127	14.4	9.60
P63046	*Sult4a1*	Sulfotransferase 4A1	m	3.9	1	1	1	1	284	33.0	5.53
P09951	*Syn1*	Synapsin-1	r	13.6	3	5	6	15	704	73.9	9.80
G3V733	*Syn2*	Synapsin II, isoform CRA_a	r	5.0	3	1	2	5	586	63.4	8.43
O70441	*Syn3*	Synapsin-3	r	2.1	2	1	1	2	579	63.3	9.44
D4ABK1	*Syngr3*	Synaptogyrin 3	r	7.5	1	1	1	4	199	21.4	9.04
P21707	*Syt1*	Synaptotagmin-1	r	13.5	7	5	5	12	421	47.4	8.41
P37805	*Tagln3*	Transgelin-3	r	7.0	2	1	1	2	199	22.5	7.33
I6L9G6	*Tardbp*	TAR DNA-binding protein	r	16.1	7	5	5	9	285	32.1	6.99
D4A4W8	*Tbxt*	T, brachyury homolog (Mouse) (Predicted), isoform CRA_b	r	3.2	3	1	1	1	436	47.4	6.99
P11983	*Tcp1*	T-complex protein 1 subunit alpha	m	14.9	7	7	7	11	556	60.4	6.16
B9EHJ3	*Tjp1*	Tight junction protein ZO-1	m	9.9	2	13	14	30	1685	188.7	6.67
Q9Z0U1	*Tjp2*	Tight junction protein ZO-2	m	7.2	1	7	7	10	1167	131.2	6.79
P40142	*Tkt*	Transketolase	m	1.3	3	1	1	1	623	67.6	7.50
P49813	*Tmod1*	Tropomodulin-1	m	4.2	3	1	1	1	359	40.4	5.10
Q9JKK7	*Tmod2*	Tropomodulin-2	m	26.5	1	1	7	59	351	39.5	5.35
P70566	*Tmod2*	Tropomodulin-2	r	25.9	1	1	7	59	351	39.5	5.43
Q6AXW2	*Tmod3*	Protein Tmod3	r	9.7	3	3	3	5	352	39.4	4.89
Q8C5G6	*Tollip*	Toll interacting protein	m	6.4	3	1	1	1	220	24.5	6.32
O88746	*Tom1*	Target of Myb protein 1	m	2.6	3	1	1	2	492	54.3	4.94

Q5SRX1	*Tom1l2*	TOM1-like protein 2	m	11.2	4	5	5	8	507	55.6	4.82
P48500	*Tpi1*	Triosephosphate isomerase	r	21.7	3	4	4	17	249	26.8	7.24
G5E8R0	*Tpm1*	Tropomyosin 1, alpha, isoform CRA_i	m	33.9	13	4	10	117	245	28.3	4.77
P97726	*Tpm3*	Tropomyosin 5	rattus sp.	40.7	4	5	12	102	248	28.9	4.75
P21107	*Tpm3*	Tropomyosin alpha-3 chain	m	30.6	2	2	10	70	284	32.8	4.72
P09495	*Tpm4*	Tropomyosin alpha-4 chain	r	30.2	7	5	9	35	248	28.5	4.69
Q3TDT0	*Trim3*	Tripartite motif-containing protein 3	m	1.7	4	1	1	1	717	78.2	8.16
Q3B8N7	*Tsc22d4*	TSC22 domain family protein 4	r	5.4	7	1	2	4	387	40.0	7.44
P68362	*Tuba1a*	Tubulin alpha-1A chain	Cg	45.5	9	5	17	667	451	50.1	5.06
P68368	*Tuba4a*	Tubulin alpha-4A chain	m	36.4	2	3	13	189	448	49.9	5.06
Q7TMM9	*Tubb2a*	Tubulin beta-2A chain	m	41.8	1	1	15	463	445	49.9	4.89
Q9CWF2	*Tubb2b*	Tubulin beta-2B chain	m	41.8	1	1	15	472	445	49.9	4.89
Q9ERD7	*Tubb3*	Tubulin beta-3 chain	m	41.8	2	4	15	381	450	50.4	4.93
Q9D6F9	*Tubb4a*	Tubulin beta-4A chain	m	33.6	1	2	12	377	444	49.6	4.88
P68372	*Tubb4b*	Tubulin beta-4B chain	m	41.8	3	1	15	405	445	49.8	4.89
P69893	*Tubb5*	Tubulin beta-5 chain	Cg	38.1	1	2	14	467	444	49.6	4.89
B2RYP8	*Tubgcp2*	A disintegrin and metalloprotease domain 8 (Predicted), isoform CRA_b	r	2.3	2	2	2	4	905	103.0	6.64
P86251	*Tufm*	Elongation factor Tu, mitochondrial (Fragments)	Ma	6.3	4	1	1	1	174	18.8	6.61
P10639	*Txn*	Thioredoxin	m	12.4	2	1	1	2	105	11.7	4.92
Q5U300	*Uba1*	Ubiquitin-like modifier-activating enzyme 1	r	6.4	4	5	5	6	1058	117.7	5.57
P62984	*Uba52*	Ubiquitin-60S ribosomal protein L40	m	44.5	12	1	6	37	128	14.7	9.83
Q80X50	*Ubap2l*	Ubiquitin-associated protein 2-like	m	2.3	1	2	2	4	1107	116.7	7.11
Q9EQX9	*Ube2n*	Ubiquitin-conjugating enzyme E2 N	r	7.2	1	1	1	4	152	17.1	6.57
B7ZBY6	*Ube2v1*	Ubiquitin-conjugating enzyme E2 variant 1 (Fragment)	m	8.0	7	1	1	1	125	13.9	5.25
Q9R0P9	*Uchl1*	Ubiquitin carboxyl-terminal hydrolase isozyme L1	m	24.2	1	3	3	6	223	24.8	5.24
P63024	*Vamp3*	Vesicle-associated membrane protein 3	m	16.5	3	1	1	2	103	11.5	8.50
P46462	*Vcp*	Transitional endoplasmic reticulum ATPase	r	10.4	2	8	8	15	806	89.3	5.26
Q9Z2L0	*Vdac1*	Voltage-dependent anion-selective channel protein 1	r	12.4	6	3	3	9	283	30.7	8.54
G3UX26	*Vdac2*	Voltage-dependent anion-selective channel protein 2 (Fragment)	m	14.5	4	2	3	6	283	30.4	7.58
Q9R1Z0	*Vdac3*	Voltage-dependent anion-selective channel protein 3	r	12.0	1	2	3	7	283	30.8	8.70
G3V8C3	*Vim*	Vimentin	r	54.3	17	25	28	753	466	53.7	5.12
G3V8A5	*Vps35*	Protein Vps35	r	2.5	2	2	2	3	796	91.7	5.44

Q5BJU7	*Wasf1*	Wiskott-Aldrich syndrome protein family member 1	r	6.3	2	3	3	6	559	61.5	6.37
G3V9M3	*Wdr47*	WD repeat domain 47	r	2.7	2	2	2	4	921	102.3	5.94
Q9ESZ0	*Xrcc1*	DNA repair protein XRCC1	r	2.2	1	1	1	1	631	68.8	6.60
P62960	*Ybx1*	Nuclease-sensitive element-binding protein 1	m	32.0	9	7	7	21	322	35.7	9.88
P35213	*Ywhab*	14-3-3 protein beta/alpha	r	33.7	5	4	7	34	246	28.0	4.88
P62259	*Ywhae*	14-3-3 protein epsilon	m	40.4	3	7	9	32	255	29.2	4.74
P61982	*Ywhag*	14-3-3 protein gamma	m	20.7	3	2	5	30	247	28.3	4.89
P68510	*Ywhah*	14-3-3 protein eta	m	22.0	3	3	5	24	246	28.2	4.89
P68254	*Ywhaq*	14-3-3 protein theta	m	35.1	3	5	7	30	245	27.8	4.78
P63101	*Ywhaz*	14-3-3 protein zeta/delta	m	34.3	3	5	7	35	245	27.8	4.79
D4AA64	*Zc3h7b*	Zinc finger CCCH type containing 7B	r	1.5	1	1	1	1	989	111.1	7.33
B1WBW3	*Zfp579*	Zinc finger protein 579	r	2.9	2	1	1	1	562	60.8	8.72
