# Supplementary material for: Comparative Proteomics Unveils LRRFIP1 as a New Player in the DAPK1 Interactome of Neurons Exposed to Oxygen and Glucose Deprivation
Source: Antioxidants (Basel). 2020 Nov 30;9(12):1202. doi: 10.3390/antiox9121202 (PMC7761126; doi:10.3390/antiox9121202)
Supplement: Supplementary file 1 [file antioxidants-09-01202-s001.zip › Table S3.pdf]

Supplementary material of the MS '**Comparative proteomics unveils LRRFIP1 as a new player in the DAPK1 interactome of neurons exposed to oxygen and glucose deprivation'**, by DeGregorio-Rocasolano et al.

Table S3. Protein partners that appear/increase in the neuronal DAPK1 interactome after exposure to OGD (abbreviations: m: mouse; r: rat; Cp: Cavia porcellus; Cla: Chinchilla lanigera; Cg: Cricetulus griseus; Clo: Cricetulus longicaudatus; Ds: Dipus sagitta; Ma: Mesocricetus aurus; m: mouse; r: rat; Sc: Spermophilus citellus; Sp: Spermophilus parryii).

Accession	Gene symbol	Description	Species	ΣCoverage	Σ# Proteins	Σ# Unique Peptides	Σ# Peptides	Σ# PSMs	# AAs	MW [kDa]	calc. pI
Q2LDP2	-	Pyruvate dehydrogenase E1 alpha (Fragment)	Sp	32.8	5	2	2	4	61	6.8	5.19
Q9CPN9	*2210010C04Rik*	RIKEN cDNA 2210010C04 gene (trypsinogen 7)	m	4.9	1	1	1	3	247	26.4	7.93
P50475	*Aars*	Alanine-tRNA ligase, cytoplasmic	r	1.5	1	1	1	1	968	106.7	5.62
P07872	*Acox1*	Peroxisomal acyl-coenzyme A oxidase 1	r	1.4	1	1	1	1	661	74.6	8.47
Q9D9S0	*Acsl5*	Acyl-CoA synthetase long-chain family member 5, isoform CRA_a	m	4.1	7	1	1	1	268	29.6	5.96
G3V9U1	*Agap1*	Centaurin, gamma 2 (Predicted)	r	1.8	2	1	1	2	669	73.7	8.25
Q8VHH5	*Agap3*	Arf-GAP with GTPase, ANK repeat and PH domain-containing protein 3	m	1.2	1	1	1	1	910	97.9	7.75
B1AXE8	*Amot*	Angiomotin (Fragment)	m	1.3	2	1	1	10	671	75.6	7.94
P0C6S7	*Anks1b*	Ankyrin repeat and sterile alpha motif domain-containing protein 1B	r	0.9	1	1	1	2	1260	139.1	6.30
D3ZUY8	*Ap2a1*	Adaptor protein complex AP-2, alpha 1 subunit (Predicted)	r	10.6	2	4	8	13	977	107.6	7.06
Q5XJY5	*Arcn1*	Coatomer subunit delta	m	2.2	2	1	1	1	511	57.2	6.21
Q6PIC6	*Atp1a3*	Sodium/potassium-transporting ATPase subunit alpha-3	m	3.7	15	3	3	4	1013	111.6	5.41
P07340	*Atp1b1*	Sodium/potassium-transporting ATPase subunit beta-1	r	3.6	3	1	1	1	304	35.2	8.65
D3Z9R8	*Atp5mpl*	ATP synthase subunit ATP5MPL, mitochondrial	r	18.3	1	1	1	1	60	6.9	9.92
G3V7L8	*Atp6v1e1*	ATPase, H+ transporting, V1 subunit E isoform 1, isoform CRA_a	r	3.5	3	1	1	1	226	26.1	8.43
O35143	*Atpif1*	ATPase inhibitor, mitochondrial	m	10.4	2	1	1	1	106	12.2	9.64
Q3TGG2	*Atxn2l*	Ataxin-2-like protein	m	1.7	2	1	1	1	994	105.4	7.97
Q5FVQ1	*C2cd4c*	C2 calcium-dependent domain-containing protein 4C	m	1.2	1	1	1	7	513	59.1	8.73

P08413	*Camk2b*	Calcium/calmodulin-dependent protein kinase type II subunit beta	r	43.4	10	10	17	227	542	60.4	7.17
Q63092	*Camkv*	CaM kinase-like vesicle-associated protein	r	21.0	2	8	8	17	504	54.1	5.54
Q8VDP4	*Ccar2*	Cell cycle and apoptosis regulator protein 2	m	1.1	1	1	1	2	922	102.9	5.25
D3ZBX9	*Ccdc92*	Coiled-coil domain-containing 92	r	5.1	1	1	1	2	314	35.1	9.38
D3ZZM9	*Ccsap*	Centriole, cilia and spindle-associated protein	r	4.8	1	1	1	1	252	28.4	9.23
B2RQQ7	*Cdc42bpb*	CDC42 binding protein kinase beta	m	1.9	3	2	2	3	1713	194.6	6.46
Q5FVI4	*Cend1*	Cell cycle exit and neuronal differentiation protein 1	r	8.7	1	1	1	1	149	15.0	8.97
Q9D9P1	*Chchd3*	Coiled-coil-helix-coiled-coil-helix domain-containing protein 3, mitochondrial	m	6.3	3	1	1	1	175	20.4	8.27
D4A7N1	*Chchd6*	Coiled-coil-helix-coiled-coil-helix domain-containing protein 6, mitochondrial	r	3.1	2	1	1	1	261	29.2	7.94
Q9D8B3	*Chmp4b*	Charged multivesicular body protein 4b	m	15.6	2	3	3	7	224	24.9	4.82
Q6Q0N0	*Clstn1*	Calsyntenin-1	r	0.7	2	1	1	1	952	106.2	5.01
A2AI78	*Cnksr2*	Connector enhancer of kinase suppressor of Ras 2	m	1.1	3	1	1	1	896	101.6	7.11
P13233	*Cnp*	2',3'-cyclic-nucleotide 3'-phosphodiesterase	r	3.3	1	1	1	1	420	47.2	8.90
P26231	*Ctnna1*	Catenin alpha-1	m	2.7	2	1	2	5	906	100.0	6.23
O35927	*Ctnnd2*	Catenin delta-2	m	3.5	5	4	4	4	1247	134.9	7.65
P00787	*Ctsb*	Cathepsin B	r	8.0	2	3	3	5	339	37.4	5.60
P11030	*Dbi*	Acyl-CoA-binding protein	r	39.1	3	2	2	6	87	10.0	8.82
Q6AY55	*Dcakd*	Dephospho-CoA kinase domain-containing protein	r	4.6	1	1	1	2	240	27.3	9.69
Q641Y8	*Ddx1*	ATP-dependent RNA helicase DDX1	r	1.5	2	1	1	1	740	82.4	7.23
Q9WTM2	*DDX6*	Probable ATP-dependent RNA helicase DDX6 (Fragment)	Cp	2.3	3	1	1	1	472	53.2	8.66
Q5RJY4	*Dhrs7b*	Dehydrogenase/reductase SDR family member 7B	r	5.2	1	1	1	1	325	35.3	9.55
D4A9D6	*Dhx9*	DEAH (Asp-Glu-Ala-His) box polypeptide 9 (Predicted)	r	1.0	2	1	1	1	1174	131.6	6.35
B1AR31	*Dlg4*	Discs, large homolog 4 (Drosophila) (Fragment)	m	4.8	3	1	1	1	248	26.5	5.00
D4A9H1	*Dlg5*	Discs, large homolog 5 (Drosophila) (Predicted)	r	1.2	1	1	1	1	1355	150.8	8.02
P63037	*Dnaja1*	DnaJ homolog subfamily A member 1	m	13.4	4	4	4	7	397	44.8	7.08
O35824	*Dnaja2*	DnaJ homolog subfamily A member 2	r	17.5	3	6	6	17	412	45.7	6.48
D4ABX4	*Dnajb6*	DnaJ homolog subfamily B member 6	r	14.2	7	3	3	6	261	29.4	4.30
Q8CIQ7	*Dock3*	Dedicator of cytokinesis protein 3	m	0.6	1	1	1	1	2027	232.8	6.99
D4A8N1	*Dpm1*	Dolichol-phosphate mannosyltransferase subunit 1	r	4.6	1	1	1	1	260	29.2	9.50
Q7M0E3	*Dstn*	Destrin	r	20.0	2	3	3	7	165	18.5	8.03
Q07139	*Ect2*	Epithelial cell-transforming sequence 2 oncogene	m	0.9	1	1	1	1	913	103.1	7.44

Q3U5H6	*Eif2s3x*	Putative uncharacterized protein (Fragment)	m	4.2	4	1	1	1	330	35.7	8.54
Q6NZJ6	*Eif4g1*	Eukaryotic translation initiation factor 4 gamma 1	m	0.8	1	1	1	2	1600	176.0	5.40
P70372	*Elavl1*	ELAV-like protein 1	m	9.2	1	3	3	6	326	36.1	9.04
Q8VEH5	*Epm2aip1*	EPM2A-interacting protein 1	m	2.2	1	1	1	1	606	70.1	5.87
Q6TXE9	*Eprs*	Glutamyl-prolyl-tRNA synthetase	r	2.2	2	2	2	3	1486	166.7	7.50
Q5RKI5	*Flii*	Flightless I homolog (Drosophila)	r	8.3	2	9	9	16	1270	144.8	6.00
Q6MG17	*Flot1*	Flotillin 1	r	3.7	3	1	1	1	352	39.7	7.42
P85845	*Fscn1*	Fascin	r	43.0	1	3	19	166	493	54.5	6.74
Q61553	*Fscn1*	Fascin	m	36.3	1	1	17	177	493	54.5	6.89
Q64467	*Gapdhs*	Glyceraldehyde-3-phosphate dehydrogenase, testis-specific	m	7.1	4	1	2	24	440	47.6	7.88
B7FAU8	*Gdi1*	Guanosine diphosphate (GDP) dissociation inhibitor 1 (Fragment)	m	20.8	4	2	2	2	149	16.9	4.83
Q9Z254	*Gipc1*	PDZ domain-containing protein GIPC1	r	15.3	4	4	4	7	333	36.1	5.91
Q5F258	*Git1*	ARF GTPase-activating protein GIT1	m	3.2	3	2	2	2	761	84.1	6.74
P08050	*Gja1*	Gap junction alpha-1 protein	r	30.1	3	7	7	44	382	43.0	8.76
P10860	*Glud1*	Glutamate dehydrogenase 1, mitochondrial	r	29.8	3	13	13	59	558	61.4	8.00
G3V6Q6	*Gna11*	Guanine nucleotide binding protein, alpha 11	r	9.8	3	1	3	5	359	42.0	6.29
P04897	*Gnai2*	Guanine nucleotide-binding protein G(i) subunit alpha-2	r	9.9	31	2	3	17	355	40.5	5.45
P16052	*GNAS*	Guanine nucleotide-binding protein G(s) subunit alpha	Clo	6.9	25	1	2	16	394	45.6	5.82
D3YZX3	*Gnb2*	Guanine nucleotide-binding protein G(I)/G(S)/G(T) subunit beta-2	m	18.2	11	1	5	18	296	32.4	6.15
G3V9X2	*Gpsm1*	G-protein signalling modulator 1 (AGS3-like, C. elegans), isoform CRA_c	r	1.0	4	1	1	1	673	74.4	6.30
O35127	*Grcc10*	Gene rich cluster C10 protein	m	22.2	1	2	2	2	126	13.2	5.14
A2AI19	*Grin1*	Glutamate [NMDA] receptor subunit zeta-1	m	0.9	9	1	1	1	901	101.3	8.27
G3V746	*Grin2b*	Glutamate [NMDA] receptor subunit epsilon-2	r	1.0	4	1	1	1	1482	165.9	6.87
P18266	*Gsk3b*	Glycogen synthase kinase-3 beta	r	2.6	3	1	1	1	420	46.7	8.78
B8YDD1	*Hax1*	HS1 binding protein variant IX	r	8.0	2	1	1	1	151	17.1	6.21
D4ADD3	*Hecw2*	HECT, C2 and WW domain-containing E3 ubiquitin protein ligase 2	r	1.0	2	1	1	1	1578	176.4	5.39
Q4U2R1	*Herc2*	E3 ubiquitin-protein ligase HERC2	m	0.7	1	3	3	6	4836	527.1	6.27
O54792	*Hes2*	Transcription factor HES-2	m	5.1	1	1	1	1	157	17.2	10.07
P13704	*HMGCS1*	Hydroxymethylglutaryl-CoA synthase, cytoplasmic	Cg	2.5	3	1	1	1	520	57.3	5.66
O35737	*Hnrnph1*	Heterogeneous nuclear ribonucleoprotein H	m	14.9	8	5	5	15	449	49.2	6.30

Q8R081	*Hnrnpl*	Heterogeneous nuclear ribonucleoprotein L	m	10.1	2	3	3	4	586	63.9	8.10
D3ZC55	*Hspa12a*	Heat shock 70kDa protein 12A (Predicted), isoform CRA_a	r	1.3	2	1	1	1	675	74.8	6.61
Q80Z01	*hspb3*	Small heat shock protein B3 (Fragment)	Ds	5.3	2	1	1	1	131	14.5	5.27
Q66HA8	*Hsph1*	Heat shock protein 105 kDa	r	2.5	3	2	2	5	858	96.4	5.55
Q8VHU4	*Ikbkap*	Elongator complex protein 1	r	0.8	1	1	1	1	1331	149.1	6.39
Q3KR86	*Immt*	Mitochondrial inner membrane protein (Fragment)	r	8.2	2	2	4	7	609	67.1	5.80
Q8CAQ8	*Immt*	Mitochondrial inner membrane protein	m	4.2	2	1	3	5	757	83.8	6.61
D3ZLZ7	*Impdh1*	Inosine-5'-monophosphate dehydrogenase 1	r	2.3	2	1	1	1	514	55.3	6.80
E9PU28	*Impdh2*	Inosine-5'-monophosphate dehydrogenase 2	r	2.3	3	1	1	1	514	55.8	7.28
D3YUD3	*Inpp4a*	Type I inositol 3,4-bisphosphate 4-phosphatase	m	1.2	3	1	1	1	939	105.5	6.98
Q7TQK1	*Ints7*	Integrator complex subunit 7	m	1.5	1	1	1	1	966	106.8	8.22
B2KF90	*Itpr2*	Inositol 1,4,5-triphosphate receptor 2	m	0.4	3	1	1	1	2668	303.8	6.47
Q3YAA9	*Kcnip3*	Calsenilin isoform 4	m	3.5	4	1	1	1	230	26.4	4.77
B1AQZ5	*Kif3a*	Kinesin family member 3A (Fragment)	m	6.0	5	1	1	1	185	21.8	8.03
G3V6L4	*Kif5c*	Kinesin family member 5C (Predicted)	r	7.1	9	6	6	9	955	109.1	6.19
Q9ERE2	*Krt2*	Keratin, type II cuticular Hb1 (Fragment)	m	2.8	5	1	1	1	390	43.7	5.20
Q9Z2T6	*Krt85*	Keratin, type II cuticular Hb5	m	2.0	1	1	1	2	507	55.7	6.42
P04642	*Ldha*	L-lactate dehydrogenase A chain	r	6.6	8	2	2	4	332	36.4	8.27
A6H5U5	*Lrrfip1*	Lrrfip1 protein	m	5.3	3	1	3	13	663	75.1	5.85
G5E8E1	*Lrrfip1*	Leucine rich repeat (In FLII) interacting protein 1, isoform CRA_e	m	8.4	3	1	3	14	428	48.9	5.57
Q4V7E8	*Lrrfip2*	Leucine-rich repeat flightless-interacting protein 2	r	23.8	2	7	8	18	437	49.7	5.95
Q8K310	*Matr3*	Matrin-3	m	7.6	3	5	5	8	846	94.6	6.25
Q8BI84	*Mia3*	Melanoma inhibitory activity protein 3	m	0.7	1	1	1	1	1930	213.5	4.75
Q5M9I6	*Mmtag2*	Multiple myeloma tumor-associated protein 2 homolog	r	3.1	2	1	1	1	260	29.3	9.85
Q5SV64	*Myh10*	Myosin, heavy polypeptide 10, non-muscle	m	52.2	17	4	108	1535	2007	232.3	5.54
Q9JLT0	*Myh10*	Myosin-10	r	52.5	16	7	110	1574	1976	228.8	5.60
B1AR69	*Myh13*	Myosin, heavy polypeptide 13, skeletal muscle	m	0.9	12	1	2	16	1938	223.4	5.57
Q6URW6	*Myh14*	Myosin-14	m	12.0	12	13	22	148	2000	228.4	5.55
Q60605	*Myl6*	Myosin light polypeptide 6	m	47.7	11	7	8	316	151	16.9	4.65
B2RWR8	*Myo6*	Myosin VI	m	23.4	5	26	27	145	1262	145.8	8.47
Q60817	*Naca*	Nascent polypeptide-associated complex subunit alpha	m	20.5	3	3	3	5	215	23.4	4.56
Q3TF41	*Nap1l1*	Nucleosome assembly protein 1-like 1, isoform CRA_d	m	7.3	5	1	2	22	368	42.7	4.55
Q5U2Z3	*Nap1l4*	Nucleosome assembly protein 1-like 4	r	9.8	3	2	3	19	386	43.9	4.68

Q8CH77	*Nav1*	Neuron navigator 1	m	2.0	1	3	3	7	1875	202.2	8.06
Q9EPN1	*Nbea*	Neurobeachin	m	0.9	1	2	2	3	2936	326.5	6.20
P19527	*Nefl*	Neurofilament light polypeptide	r	31.2	4	14	16	115	542	61.3	4.65
G3V7S2	*Nefm*	Neurofilament 3, medium	r	25.9	5	18	21	138	845	95.7	4.79
G3V8F8	*Nes*	Nestin, isoform CRA_b	r	8.0	4	13	13	25	1893	208.8	4.32
Q9QUL6	*Nsf*	Vesicle-fusing ATPase	r	27.3	3	17	17	42	744	82.6	6.99
Q9Z0W3	*Nup160*	Nuclear pore complex protein Nup160	m	0.7	1	1	1	2	1402	158.1	5.52
A0JPJ7	*Ola1*	Obg-like ATPase 1	r	3.3	2	1	1	1	396	44.5	7.77
Q61990	*Pcbp2*	Poly(rC)-binding protein 2	m	9.9	4	2	3	7	362	38.2	6.79
G3V9G0	*Pcdhb21*	Protocadherin beta 21	r	2.5	1	1	1	1	757	82.8	4.79
P31044	*Pebp1*	Phosphatidylethanolamine-binding protein 1	r	7.0	1	1	1	1	187	20.8	5.80
Q9DBD5	*Pelp1*	Proline-, glutamic acid- and leucine-rich protein 1	m	1.4	2	1	1	1	1123	118.0	4.36
Q9EPC6	*Pfn2*	Profilin-2 [PROF2_RAT]	r	10.0	2	1	1	1	140	15.0	6.99
O08651	*Phgdh*	D-3-phosphoglycerate dehydrogenase	r	4.9	2	2	2	3	533	56.5	6.71
Q6S3A1	*Plec*	Plectin	r	2.2	11	9	9	11	4451	506.4	5.68
Q7TQG1	*Plekha6*	Pleckstrin homology domain-containing family A member 6	m	1.0	1	1	1	1	1173	131.3	8.97
P37230	*Ppara*	Peroxisome proliferator-activated receptor alpha	r	2.6	1	1	1	1	468	52.3	6.25
P60469	*Ppfia3*	Liprin-alpha-3	m	1.0	3	1	1	1	1043	116.2	5.96
Q80Y24	*Prickle2*	Prickle-like protein 2	m	1.1	2	1	1	1	845	95.7	7.27
P68404	*Prkcb*	Protein kinase C beta type	m	4.3	4	3	3	7	671	76.7	7.01
P09216	*Prkce*	Protein kinase C epsilon type	r	7.3	2	4	4	4	737	83.4	6.95
Q6QN02	*Prox1*	Homeobox prox 1 (Fragment)	Cla	11.7	5	2	2	4	145	16.0	6.19
O08618	*Prpsap2*	Phosphoribosyl pyrophosphate synthase-associated protein 2	r	2.4	2	1	1	2	369	40.8	7.17
Q2PFD7	*Psd3*	PH and SEC7 domain-containing protein 3	m	2.2	2	2	2	2	1037	114.7	6.24
P62192	*Psmc1*	26S protease regulatory subunit 4	m	2.5	1	1	1	1	440	49.2	6.21
G3V7L6	*Psmc2*	26S protease regulatory subunit 7	r	8.6	4	3	3	4	433	48.6	5.95
P62334	*Psmc6*	26S protease regulatory subunit 10B	m	2.8	2	1	1	1	389	44.1	7.49
F1LMZ8	*Psmd11*	26S proteasome non-ATPase regulatory subunit 11	r	9.2	3	3	3	3	422	47.4	6.48
O35226	*Psmd4*	26S proteasome non-ATPase regulatory subunit 4	m	4.0	3	1	1	2	376	40.7	4.79
Q3UEB3	*Puf60*	Poly(U)-binding-splicing factor PUF60	m	1.8	1	1	1	1	564	60.2	5.29
G3X8R5	*Qrich1*	Glutamine-rich protein 1	m	1.2	2	1	1	1	777	86.5	5.87
P0C643	*Rasgrp2*	RAS guanyl-releasing protein 2	r	2.3	2	1	1	1	608	69.2	7.68
Q8R4X3	*Rbm12*	RNA-binding protein 12	m	1.4	2	1	1	7	992	102.7	8.32
Q3TTW9	*Rgs12*	Regulator of G-protein-signaling 12	m	2.8	5	1	1	1	499	54.8	8.65

Q9QUI0	*Rhoa*	Transforming protein RhoA	m	11.9	5	2	2	5	193	21.8	6.10
Q9JIR4	*Rims1*	Regulating synaptic membrane exocytosis protein 1	r	3.9	2	4	4	6	1615	179.5	9.58
P13832	*Rlc-a*	Myosin regulatory light chain RLC-A	r	40.1	5	6	6	269	172	19.9	4.81
Q91YL2	*Rnf126*	RING finger protein 126	m	5.4	2	1	1	1	313	34.1	5.17
Q9CZM2	*Rpl15*	60S ribosomal protein L15	m	22.6	2	4	4	12	204	24.1	11.62
P24049	*Rpl17*	60S ribosomal protein L17	r	13.0	4	2	2	2	184	21.4	10.18
P12001	*Rpl18*	60S ribosomal protein L18	r	21.3	3	3	3	9	188	21.6	11.78
P62717	*Rpl18a*	60S ribosomal protein L18a	m	11.9	1	2	2	5	176	20.7	10.71
P62889	*Rpl30*	60S ribosomal protein L30	m	40.9	1	3	3	7	115	12.8	9.63
D4AAZ6	*Rpl37a*	60S ribosomal protein L37a	r	23.6	2	2	2	4	72	8.1	10.37
P21533	*Rpl6*	60S ribosomal protein L6	r	13.1	3	4	4	8	298	33.5	10.74
D3YVE6	*Rpl7a-ps10*	Ribosomal protein L7a, pseudogene 10 protein	m	14.7	5	3	3	7	266	30.0	10.45
G3UW34	*Rpl9-ps6*	Ribosomal protein L9, pseudogene 6 protein	m	11.5	3	2	2	4	192	21.8	9.88
P62842	*RPS15*	40S ribosomal protein S15	Ma	22.1	1	3	3	4	145	17.0	10.39
Q8C1L7	*Rps21*	40S ribosomal protein S21	m	12.4	3	1	1	2	81	8.9	8.51
Q6ZWU9	*Rps27*	40S ribosomal protein S27	m	25.0	3	2	2	7	84	9.5	9.45
P47961	*RPS4*	40S ribosomal protein S4	Cg	31.6	4	8	8	24	263	29.6	10.15
P62242	*Rps8*	40S ribosomal protein S8	m	24.5	4	4	4	9	208	24.2	10.32
D3YTT7	*Rpsa-ps10*	40S ribosomal protein SA	m	16.6	4	4	4	11	295	32.8	4.93
Q9JKY0	*Rqcd1*	Cell differentiation protein RCD1 homolog	m	3.7	1	1	1	1	299	33.6	8.03
Q5FVJ0	*Rufy3*	Protein RUFY3	r	15.4	2	6	6	10	469	52.9	5.49
P60122	*Ruvbl1*	RuvB-like 1	m	2.9	1	1	1	2	456	50.2	6.42
O08804	*Serpinb6b*	Serine (or cysteine) peptidase inhibitor, clade B, member 6B	m	4.0	1	1	1	4	377	42.5	5.26
A0JLN0	*Sf3b1*	Splicing factor 3b, subunit 1 (Fragment)	m	3.8	3	1	1	1	496	54.4	6.10
Q8BXU5	*Sh3gl2*	Endophilin-A1	m	2.9	6	1	1	1	245	27.9	5.77
D3YZU1	*Shank1*	SH3 and multiple ankyrin repeat domains protein 1	m	0.5	2	1	1	1	2167	226.2	8.34
Q5FVG4	*Slc25a22*	Solute carrier family 25 (Mitochondrial carrier, glutamate), member 22	r	3.9	3	1	1	1	229	24.6	8.60
O35413	*Sorbs2*	Sorbin and SH3 domain-containing protein 2	r	0.8	1	1	1	3	1196	134.0	8.46
O08623	*Sqstm1*	Sequestosome-1	r	3.6	1	1	1	1	439	47.7	5.17
P21707	*Syt1*	Synaptotagmin-1	r	12.8	7	5	5	10	421	47.4	8.41
Q3TWL2	*Tmem55b*	Transmembrane protein 55B	m	2.5	2	1	1	1	284	30.0	8.82
Q3TC52	*Trim46*	Tripartite motif-containing protein 46	m	4.3	2	3	3	6	541	60.7	8.63
Q3B8N7	*Tsc22d4*	TSC22 domain family protein 4	r	7.8	2	2	2	2	387	40.0	7.44

Q8BJG7	*Ttll1*	Probable tubulin polyglutamylase TTLL1	m	4.0	3	1	1	2	297	34.9	9.26
Q9ERD7	*Tubb3*	Tubulin beta-3 chain	m	42.0	2	5	16	631	450	50.4	4.93
Q9D6F9	*Tubb4a*	Tubulin beta-4A chain	m	35.8	1	2	14	654	444	49.6	4.88
P83887	*Tubg1*	Tubulin gamma-1 chain	m	2.0	2	1	1	1	451	51.1	6.02
Q6P5E4	*Uggt1*	UDP-glucose:glycoprotein glucosyltransferase 1	m	0.5	2	1	1	1	1551	176.3	5.62
Q68FY0	*Uqcrc1*	Cytochrome b-c1 complex subunit 1, mitochondrial	r	2.5	2	1	1	1	480	52.8	5.88
D3ZC84	*Usp9x*	Ubiquitin carboxyl-terminal hydrolase	r	0.4	3	1	1	1	2547	289.3	5.78
Q9WV55	*Vapa*	Vesicle-associated membrane protein-associated protein A	m	10.8	2	2	2	2	249	27.8	8.40
G3V8C3	*Vim*	Vimentin	r	54.3	17	25	28	753	466	53.7	5.12
O08700	*Vps45*	Vacuolar protein sorting-associated protein 45	r	2.1	3	1	1	1	570	64.9	8.25
G3V9M3	*Wdr47*	WD repeat domain 47	r	3.9	2	3	3	7	921	102.3	5.94
Q9ERH3	*Wdr7*	WD repeat-containing protein 7	r	1.6	3	2	2	2	1488	163.1	7.02
Q4QR85	*Wdr77*	Methylosome protein 50	r	4.4	2	1	1	1	342	37.1	5.27
Q6NXJ0	*Wwc2*	WW domain-containing protein 2	m	1.5	2	1	1	1	1187	132.5	5.71
F1LM93	*Yes1*	Tyrosine-protein kinase Yes	r	2.2	3	1	1	1	541	60.6	6.64
