# Supplementary material for: Comparative Proteomics Unveils LRRFIP1 as a New Player in the DAPK1 Interactome of Neurons Exposed to Oxygen and Glucose Deprivation
Source: Antioxidants (Basel). 2020 Nov 30;9(12):1202. doi: 10.3390/antiox9121202 (PMC7761126; doi:10.3390/antiox9121202)
Supplement: Supplementary file 1 [file antioxidants-09-01202-s001.zip › Table S4.pdf]

Supplementary material of the MS **'Comparative proteomics unveils LRRFIP1 as a new player in the DAPK1 interactome of neurons exposed to oxygen and glucose deprivation'**, by DeGregorio-Rocasolano et al.

Table S4. Protein partners that disappear/decrease in the neuronal DAPK1 interactome after exposure to OGD (abbreviations: m: mouse; r: rat; Cp: Cavia porcellus; Cla: Chinchilla lanigera; Cg: Cricetulus griseus; Clo: Cricetulus longicaudatus; Hg: Heterocephalus glaber; Ma: Mesocricetus aurus; m: mouse; Mg: Myodes glareolus; r: rat; Sc: Spermophilus citellus; St: Spermophilus tridecemlineatus; Tm: Tokudaia muenninki).

Accession	Gene symbol	Description	Species	ΣCoverage	Σ# Proteins	Σ# Unique Peptides	Σ# Peptides	Σ# PSMs	# AAs	MW [kDa]	calc. pI
B7ZCU1	*Abi1*	Abl-interactor 1	m	4.1	11	1	1	1	388	42.5	6.20
Q6KC51	*Ablim2*	Actin-binding LIM protein 2	r	1.5	2	1	1	1	612	68.0	7.94
Q3TS02	*Acly*	ATP citrate lyase	m	1.6	6	1	1	1	491	53.7	7.06
Q99KI0	*Aco2*	Aconitate hydratase, mitochondrial	m	1.9	2	1	1	1	780	85.4	7.93
Q6GMN8	*Actn1*	Actn1 protein	r	21.3	7	3	17	65	887	102.5	5.48
Q9JI91	*Actn2*	Alpha-actinin-2	m	9.1	1	1	7	38	894	103.8	5.45
Q9QXQ0	*Actn4*	Alpha-actinin-4	r	42.7	4	19	33	134	911	104.8	5.44
Q5M9F7	*Actr10*	ARP10 actin-related protein 10 homolog (S. cerevisiae)	r	1.7	2	1	1	1	417	46.2	7.37
Q3UHD9	*Agap2*	Arf-GAP with GTPase, ANK repeat and PH domain-containing protein 2	m	1.5	2	1	1	3	1186	124.4	9.89
Q70FJ1	*Akap9*	A-kinase anchor protein 9	m	1.5	2	5	5	5	3797	435.9	5.03
Q9JHE0	*Akap9*	A-kinase anchor protein 9 (Fragment)	r	3.8	1	1	1	2	395	45.1	4.64
O70473	*AKR1A1*	Alcohol dehydrogenase [NADP(+)] (Fragment)	Cg	8.3	3	1	1	2	228	25.4	6.28
D3ZXH7	*Alyref*	Aly/REF export factor	r	11.1	5	2	2	4	189	19.9	9.86
D4A9E7	*Ankef1*	Ankyrin repeat and EF-hand domain-containing 1 (Predicted)	r	1.2	2	1	1	1	775	86.8	8.02
P62743	*Ap2s1*	AP-2 complex subunit sigma	m	10.6	1	2	2	4	142	17.0	6.18
G3V8Q9	*Apc*	Adenomatosis polyposis coli, isoform CRA_a	r	1.5	4	3	3	6	2842	310.3	7.37
P08226	*Apoe*	Apolipoprotein E	m	10.6	5	3	3	5	311	35.8	5.68
Q6DFV3	*Arhgap21*	Rho GTPase-activating protein 21	m	1.3	3	2	2	3	1944	215.6	7.64
Q4KLF8	*Arpc5*	Actin-related protein 2/3 complex subunit 5	r	20.5	4	2	2	5	151	16.3	5.67
P19511	*Atp5f1*	ATP synthase subunit b, mitochondrial	r	4.7	1	1	1	3	256	28.9	9.36
Q06647	*Atp5o*	ATP synthase subunit O, mitochondrial	r	6.6	2	1	1	1	213	23.4	10.02

A2A5A2	*Atp6v0a1*	ATPase, H+ transporting, lysosomal V0 subunit A1, isoform CRA_a	m	7.8	6	5	5	10	832	95.6	6.77
D4A133	*Atp6v1a*	ATPase H+ transporting V1 subunit A	r	14.9	3	8	8	12	617	68.2	5.58
Q5U2U8	*Bag3*	Bcl2-associated athanogene 3	r	3.0	1	1	1	5	574	61.5	7.03
B1AZ45	*Baiap2*	Brain-specific angiogenesis inhibitor 1-associated protein 2	m	2.7	7	1	1	1	482	53.2	8.98
Q05175	*Basp1*	Brain acid soluble protein 1	r	41.4	1	4	6	18	220	21.8	4.51
Q3ZB98	*Bcas1*	Breast carcinoma-amplified sequence 1 homolog (Fragment)	r	3.2	1	1	1	1	555	58.6	5.73
P70562	*Bhlha15*	Class A basic helix-loop-helix protein 15	r	4.6	2	1	1	1	197	22.2	11.15
Q62717	*Cadps*	Calcium-dependent secretion activator 1	r	2.1	2	2	2	4	1289	146.2	5.82
Q80VC9	*Camsap3*	Calmodulin-regulated spectrin-associated protein 3	m	1.1	1	1	1	1	1252	135.1	8.43
P35565	*Canx*	Calnexin	r	2.0	1	1	1	1	591	67.2	4.63
Q5XIM9	*Cct2*	T-complex protein 1 subunit beta	r	3.7	3	2	2	4	535	57.4	6.46
P80317	*Cct6a*	T-complex protein 1 subunit zeta	m	10.4	4	4	4	4	531	58.0	7.08
D4AC23	*Cct7*	T-complex protein 1 subunit eta	r	13.8	2	5	5	5	544	59.6	8.00
D4ACB8	*Cct8*	Chaperonin subunit 8 (Theta) (Predicted), isoform CRA_a	r	29.9	2	15	15	18	548	59.6	5.54
Q6A065	*Cep170*	Centrosomal protein of 170 kDa	m	5.5	1	7	7	9	1588	174.9	7.17
P60826	*CIRBP*	Cold-inducible RNA-binding protein	Cg	20.9	2	3	3	4	172	18.6	9.61
A2AGT5	*Ckap5*	Cytoskeleton-associated protein 5	m	0.5	1	1	1	1	2032	225.5	7.96
Q99JD4	*Clasp2*	CLIP-associating protein 2	r	15.6	5	18	18	34	1286	140.6	8.57
Q922J3	*Clip1*	CAP-Gly domain-containing linker protein 1	m	13.2	2	11	15	26	1391	155.7	5.24
B1AWE0	*Clta*	Clathrin light chain A	m	7.9	8	2	2	2	216	23.5	4.50
P12960	*Cntn1*	Contactin-1	m	1.0	2	1	1	1	1020	113.3	6.16
P23514	*Copb1*	Coatomer subunit beta	r	1.5	2	1	1	2	953	106.9	5.96
O35142	*Copb2*	Coatomer subunit beta'	r	1.0	2	1	1	1	905	102.5	5.27
P11240	*Cox5a*	Cytochrome c oxidase subunit 5A, mitochondrial	r	10.3	2	1	1	2	146	16.1	6.54
Q5PR69	*Crad*	Capping protein inhibiting regulator of actin dynamics	m	5.1	2	5	5	11	1207	132.2	5.40
Q9JJ76	*Csnk1e*	Casein kinase 1 epsilon	r	3.1	3	1	1	1	416	47.3	9.66
Q9D0E8	*Csnk2a1*	Casein kinase II, alpha 1 polypeptide, isoform CRA_b	m	23.4	5	4	4	8	248	29.5	7.94
P67871	*Csnk2b*	Casein kinase II subunit beta	m	15.8	2	3	3	3	215	24.9	5.55
D4A1B8	*Dctn3*	Dynactin subunit 3	r	9.7	2	2	2	3	186	21.1	5.71
D3Z6H3	*Dctn6*	Dynactin 6, isoform CRA_b	m	5.4	3	1	1	1	185	20.0	6.38
A2ADY9	*Ddi2*	Protein DDI1 homolog 2	m	15.0	1	4	4	13	399	44.6	5.05
I0J0A0	*Ddx3y*	DEAD (Asp-Glu-Ala-Asp) box polypeptide 3, Y-linked (Fragment)	Tm	5.2	8	3	3	4	559	62.8	7.30

G3V792	*Dync1i1*	Cytoplasmic dynein 1 intermediate chain 1	r	4.0	2	2	2	3	643	72.6	5.12
A2BFF8	*Dync1i2*	Cytoplasmic dynein 1 intermediate chain 2	m	5.2	11	2	2	2	611	68.2	5.29
G3V7G0	*Dync1li1*	Cytoplasmic dynein 1 light intermediate chain 1	r	23.3	3	10	10	34	523	56.6	6.29
Q5D023	*Dync1li2*	Cytoplasmic dynein 1 light intermediate chain 2	r	14.8	3	6	6	12	492	54.1	6.16
P62627	*Dynlrb1*	Dynein light chain roadblock-type 1	m	12.5	2	1	1	5	96	11.0	7.25
G3V771	*Edn2*	Endothelin 2	r	6.3	2	1	1	1	176	19.5	9.86
B5DEN5	*Eef1b2*	Eukaryotic translation elongation factor 1 beta 2	r	6.7	3	1	1	1	225	24.7	4.72
G3V732	*Eef1d*	Elongation factor 1-delta	r	4.7	3	1	1	1	257	28.7	4.97
Q68FR6	*Eef1g*	Elongation factor 1-gamma	r	3.4	2	2	2	2	437	50.0	6.74
Q6ZWX6	*Eif2s1*	Eukaryotic translation initiation factor 2 subunit 1	m	3.8	1	1	1	1	315	36.1	5.08
P04764	*Eno1*	Alpha-enolase	r	11.8	15	4	4	6	434	47.1	6.57
D4A361	*Epb4.1l2*	Erythrocyte membrane protein band 4.1-like 2	r	1.9	3	1	1	2	823	91.1	5.49
P84089	*Erh*	Enhancer of rudimentary homolog	m	26.9	2	2	2	4	104	12.3	5.92
B0BMZ1	*Fam241b*	Family with sequence similarity 241 member B	r	15.8	1	1	1	3	120	13.2	11.41
Q3TJZ6	*Fam98a*	Family with sequence similarity 98, member A protein	m	2.7	2	1	1	1	515	55.0	8.95
P12785	*Fasn*	Fatty acid synthase	r	4.3	2	9	9	12	2505	272.5	6.39
G3V6L9	*Fkbp3*	Peptidyl-prolyl cis-trans isomerase	r	4.9	2	1	1	1	224	25.2	9.28
B7FAV1	*Flna*	Filamin, alpha (Fragment)	m	0.6	5	1	1	4	2583	274.5	5.97
P97855	*G3bp1*	Ras GTPase-activating protein-binding protein 1	m	3.9	1	1	1	1	465	51.8	5.59
P60521	*Gabarapl2*	Gamma-aminobutyric acid receptor-associated protein-like 2	m	35.9	1	4	4	7	117	13.7	8.10
P13264	*Gls*	Glutaminase kidney isoform, mitochondrial	r	17.8	2	8	8	21	674	74.0	7.87
P09606	*Glul*	Glutamine synthetase	r	3.0	1	1	1	1	373	42.2	7.08
B9EJU1	*Gm1141*	Gene model 1141, (NCBI)	m	2.7	1	1	1	1	475	52.2	4.82
G3XA41	*Gm6055*	MCG50219	m	22.4	4	1	3	20	125	14.6	7.50
P35802	*Gpm6a*	Neuronal membrane glycoprotein M6-a	m	9.0	2	2	2	4	278	31.1	5.27
P23785	*Grn*	Granulins	r	2.7	4	1	1	2	588	63.3	6.47
Q02874	*H2afy*	Core histone macro-H2A.1	r	2.4	3	1	1	1	371	39.5	9.79
P02301	*H3f3c*	Histone H3.3C	m	11.8	7	2	2	3	136	15.3	11.14
Q923W4	*Hdgfrp3*	Hepatoma-derived growth factor-related protein 3	r	15.4	2	2	3	4	202	22.4	8.40
P43275	*Hist1h1a*	Histone H1.1	m	9.4	2	1	2	13	213	21.8	10.93
D3ZBN0	*Hist1h1b*	Histone H1.5 [H15_RAT]	r	14.4	2	3	3	20	222	22.6	10.96
P15864	*Hist1h1c*	Histone H1.2	m	16.0	7	4	5	57	212	21.3	11.00
Q00715	*Hist1h2b1*	Histone H2B type 1	r	29.6	22	5	5	23	125	14.0	10.36
Q8K585	*Hmga1*	High mobility group protein HMG-I/HMG-Y	r	23.4	1	3	3	7	107	11.7	10.32

O88791	*Hmga2*	High mobility group AT-hook 2	r	12.2	1	1	1	1	107	11.7	10.59
P18608	*Hmgn1*	Non-histone chromosomal protein HMG-14	m	15.6	2	1	1	1	96	10.1	9.76
P09602	*Hmgn2*	Non-histone chromosomal protein HMG-17	m	16.7	5	1	1	3	90	9.4	9.99
Q66H40	*Hmgn3*	High mobility group nucleosome-binding domain-containing protein 3	r	15.8	1	1	1	2	95	10.2	10.10
Q9CX86	*Hnrnpa0*	Heterogeneous nuclear ribonucleoprotein A0	m	11.5	1	2	2	2	305	30.5	9.31
Q9Z204	*Hnrnpc*	Heterogeneous nuclear ribonucleoproteins C1/C2	m	4.2	1	1	1	1	313	34.4	5.05
O88844	*Idh1*	Isocitrate dehydrogenase [NADP] cytoplasmic	m	3.1	4	1	1	5	414	46.6	7.17
Q9CXY6	*Ilf2*	Interleukin enhancer-binding factor 2	m	10.8	2	3	3	5	390	43.0	5.26
Q5SYL3	*Kiaa0100*	Protein KIAA0100	m	0.4	1	1	1	1	2234	254.3	7.34
Q6IG00	*Krt4*	Keratin, type II cytoskeletal 4	r	3.0	8	0	2	6	536	57.6	7.64
Q3UH68	*Limch1*	LIM and calponin homology domains-containing protein 1	m	8.1	1	7	7	8	1057	118.1	5.48
G3V7U4	*Lmnb1*	Lamin-B1	r	8.4	3	4	4	4	587	66.6	5.16
Q91VR7	*Map1lc3a*	Microtubule-associated proteins 1A/1B light chain 3A	m	23.1	1	1	3	30	121	14.3	8.68
P0C5W1	*Map1s*	Microtubule-associated protein 1S	r	1.3	1	1	1	1	972	102.7	7.11
P97820	*Map4k4*	Mitogen-activated protein kinase kinase kinase kinase 4	m	1.7	15	1	2	3	1233	140.5	7.47
P63085	*Mapk1*	Mitogen-activated protein kinase 1	m	2.5	3	1	1	1	358	41.2	6.98
Q61166	*Mapre1*	Microtubule-associated protein RP/EB family member 1	m	14.6	2	3	3	5	268	30.0	5.22
Q9Z2D6	*Mecp2*	Methyl-CpG-binding protein 2	m	3.9	2	1	1	4	484	52.3	9.96
G3V9F3	*Mprip*	Myosin phosphatase Rho-interacting protein	r	3.8	5	3	3	3	1029	116.9	6.21
O35821	*Mybbp1a*	Myb-binding protein 1A	r	0.6	1	1	1	1	1344	152.2	8.95
Q63355	*Myo1c*	Unconventional myosin-Ic	r	2.0	2	2	2	4	1044	119.7	9.39
P85969	*Napb*	Beta-soluble NSF attachment protein	r	4.4	2	1	1	2	297	33.4	5.47
P13383	*Ncl*	Nucleolin	r	4.9	5	3	3	4	713	77.1	4.74
Q9CQF3	*Nudt21*	Cleavage and polyadenylation specificity factor subunit 5	m	11.9	1	2	2	3	227	26.2	8.82
Q5XI78	*Ogdh*	2-oxoglutarate dehydrogenase, mitochondrial	r	1.2	2	1	1	2	1023	116.2	6.77
Q5DJU3	*ORC3*	Origin recognition complex subunit 3	Sc	1.1	1	1	1	1	713	82.3	8.06
P29341	*Pabpc1*	Polyadenylate-binding protein 1	m	9.0	13	5	5	9	636	70.6	9.50
P51583	*Paics*	Multifunctional protein ADE2	r	2.6	2	1	1	1	425	47.1	7.69
Q9WUJ3	*Pde4dip*	Myomegalin	r	1.0	3	2	2	2	2324	261.9	5.40
P47857	*Pfkm*	6-phosphofructokinase, muscle type	m	1.4	3	1	1	1	780	85.2	8.00
P09411	*Pgk1*	Phosphoglycerate kinase 1	m	2.6	3	1	1	1	417	44.5	7.90
P86220	*PHB*	Prohibitin (Fragments)	Ma	23.0	4	2	2	2	87	10.0	4.60
Q66WT9	*Picalm*	Clathrin-assembly lymphoid myeloid leukemia protein	r	4.5	4	1	2	2	597	64.7	9.01

O88377	*Pip4k2b*	Phosphatidylinositol 5-phosphate 4-kinase type-2 beta	r	3.4	2	1	1	1	416	47.2	7.52
P11980	*Pkm*	Pyruvate kinase isozymes M1/M2	r	4.0	2	2	2	2	531	57.8	7.06
A2BGI8	*Ppih*	Peptidyl-prolyl cis-trans isomerase (Fragment)	m	8.9	2	1	1	1	158	17.2	8.34
Q10728	*Ppp1r12a*	Protein phosphatase 1 regulatory subunit 12A	r	5.5	5	4	4	6	1032	115.2	5.47
P62715	*Ppp2cb*	Serine/threonine-protein phosphatase 2A catalytic subunit beta isoform	m	11.0	2	3	3	3	309	35.6	5.43
D3ZHI9	*Ppp2r5e*	Protein Ppp2r5e	r	2.8	7	1	1	1	429	50.3	8.16
Q63810	*Ppp3r1*	Calcineurin subunit B type 1 GN=Ppp3r1	m	5.9	1	1	1	2	170	19.3	4.81
P35700	*Prdx1*	Peroxiredoxin-1	m	21.6	8	3	3	5	199	22.2	8.12
P35704	*Prdx2*	Peroxiredoxin-2	r	23.7	3	5	5	10	198	21.8	5.59
G3V7I0	*Prdx3*	Peroxiredoxin 3	r	4.3	3	1	1	1	257	28.3	7.55
P12369	*Prkar2b*	cAMP-dependent protein kinase type II-beta regulatory subunit	r	3.9	1	1	1	3	416	46.1	4.98
G3V7J2	*Prkra*	Interferon-inducible double stranded RNA-dependent protein kinase activator A	r	11.8	3	3	3	6	313	34.4	8.56
Q566D6	*Psip1*	PC4 and SFRS1-interacting protein	r	15.7	5	3	4	6	331	37.3	9.17
D3ZGY1	*Pym1*	PYM homolog 1, exon junction complex-associated factor	r	5.4	1	1	1	1	203	22.7	9.64
Q8R361	*Rab11fip5*	Rab11 family-interacting protein 5	m	2.8	1	1	1	3	645	69.5	9.07
P62827	*Ran*	GTP-binding nuclear protein Ran	m	15.7	5	3	3	3	216	24.4	7.49
Q8C2Q3	*Rbm14*	RNA-binding protein 14	m	1.5	1	1	1	1	669	69.4	9.67
P62746	*Rhob*	Rho-related GTP-binding protein RhoB	m	6.1	1	1	1	1	196	22.1	5.24
P53026	*Rpl10a*	60S ribosomal protein L10a	m	18.9	4	3	3	7	217	24.9	9.98
O09167	*Rpl21*	60S ribosomal protein L21	m	18.1	7	2	2	2	160	18.6	10.49
P67984	*Rpl22*	60S ribosomal protein L22	m	18.8	2	2	2	8	128	14.7	9.19
P25886	*Rpl29*	60S ribosomal protein L29	r	12.2	4	2	2	3	156	17.3	11.78
P21531	*Rpl3*	60S ribosomal protein L3	r	3.0	3	1	1	1	403	46.1	10.21
Q6QMZ5	*Rpl35*	Ribosomal protein L35 (Fragment)	Cla	9.9	2	1	1	1	101	11.8	11.30
Q9D8E6	*Rpl4*	60S ribosomal protein L4	m	4.5	3	2	2	2	419	47.1	11.00
P09895	*Rpl5*	60S ribosomal protein L5	r	9.1	3	2	2	4	297	34.4	9.74
Q921R2	*Rps13*	40S ribosomal protein S13	m	22.1	3	3	3	8	140	16.1	10.71
P14131	*Rps16*	40S ribosomal protein S16	m	17.8	2	3	3	10	146	16.4	10.21
P62298	*RPS23*	40S ribosomal protein S23	Cla	8.4	1	1	1	1	143	15.8	10.49
Q6QMZ1	*Rps26*	40S ribosomal protein S26 (Fragment)	Cla	8.2	3	1	1	1	110	12.3	10.81
Q9CXW7	*Rps9*	40S ribosomal protein S9	m	6.5	2	1	1	1	139	16.6	10.80

P62071	*Rras2*	Ras-related protein R-Ras2	m	5.9	1	1	1	1	204	23.4	6.01
Q99LF4	*Rtcb*	tRNA-splicing ligase RtcB homolog	m	9.9	1	4	4	5	505	55.2	7.23
Q7M6W1	*Rtn1*	Reticulon	m	15.4	5	3	3	7	208	23.5	8.91
D4A1U2	*Rtn3*	Reticulon 3, isoform CRA_a	r	4.3	3	1	1	3	256	27.4	8.24
Q8C8N2	*Scai*	Suppressor of cancer cell invasion protein	m	1.8	1	1	1	3	606	70.2	8.60
Q80UF4	*Sdccag8*	Serologically defined colon cancer antigen 8 homolog	m	1.3	1	1	1	1	717	82.9	6.52
D3ZDY1	*Sept3*	Neuronal-specific septin-3	r	3.0	2	1	1	1	337	38.7	6.81
B5DFG5	*Sept6*	Septin 6	r	3.3	1	1	1	1	427	48.7	6.67
P63209	*SKP1*	S-phase kinase-associated protein 1	Cp	20.9	2	4	4	5	163	18.6	4.54
G3V6A2	*Slain2*	Protein Slain2	r	2.3	1	1	1	1	607	65.3	9.72
Q8JZR4	*Slc1a7*	Excitatory amino acid transporter 5	m	1.6	1	1	1	1	559	60.1	6.04
Q9ET64	*Smpd2*	Sphingomyelin phosphodiesterase 2	r	4.0	1	1	1	1	422	47.6	7.08
Q61548	*Snap91*	Clathrin coat assembly protein AP180	m	4.0	4	2	3	5	901	91.8	4.88
B0BN51	*Snrpb*	Small nuclear ribonucleoprotein-associated protein	r	10.0	5	2	2	2	231	23.6	10.90
P62320	*Snrpd3*	Small nuclear ribonucleoprotein Sm D3	m	15.1	1	2	2	4	126	13.9	10.32
Q9D0T1	*Snu13*	NHP2-like protein 1	m	9.4	1	1	1	1	128	14.2	8.46
E1U8D0	*Soga1*	Suppressor of glucose, autophagy-associated protein 1	m	0.6	2	1	1	1	1418	159.1	6.46
Q2KN98	*Specc1l*	Cytospin-A	m	3.1	2	3	3	3	1118	124.4	5.76
Q4V8J7	*Spin1*	Spindlin-1	r	5.0	2	1	1	1	262	29.6	6.96
O70559	*Sprr2h*	Small proline-rich protein 2H	m	41.7	7	1	1	1	108	11.7	7.83
Q9QWN8	*Sptbn2*	Spectrin beta chain, non-erythrocytic 2	r	26.1	5	41	49	133	2388	270.9	5.83
Q66H19	*Srfbp1*	Serum response factor-binding protein 1	r	3.2	1	1	1	1	442	49.2	9.61
A2A5R8	*Stau1*	Double-stranded RNA-binding protein Staufen homolog 1	m	2.7	7	1	1	1	485	53.7	9.55
D3ZDD7	*Strbp*	Spermatid perinuclear RNA binding protein, isoform CRA_a	r	1.3	5	1	1	1	672	73.7	8.68
P11031	*Sub1*	Activated RNA polymerase II transcriptional coactivator p15	m	15.8	2	2	2	2	127	14.4	9.60
P63046	*Sult4a1*	Sulfotransferase 4A1	m	3.9	1	1	1	1	284	33.0	5.53
O70441	*Syn3*	Synapsin-3	r	2.1	2	1	1	1	579	63.3	9.44
P37805	*Tagln3*	Transgelin-3	r	7.0	2	1	1	1	199	22.5	7.33
D4A4W8	*Tbxt*	T, brachyury homolog (Mouse) (Predicted), isoform CRA_b	r	3.2	3	1	1	1	436	47.4	6.99
P11983	*Tcp1*	T-complex protein 1 subunit alpha	m	14.9	7	7	7	11	556	60.4	6.16
P40142	*Tkt*	Transketolase	m	1.3	3	1	1	1	623	67.6	7.50
P49813	*Tmod1*	Tropomodulin-1	m	6.1	3	1	2	2	359	40.4	5.10
Q8C5G6	*Tollip*	Toll interacting protein	m	6.4	3	1	1	1	220	24.5	6.32
Q3TDT0	*Trim3*	Tripartite motif-containing protein 3	m	2.1	4	1	1	1	717	78.2	8.16

P10639	*Txn*	Thioredoxin	m	12.4	2	1	1	1	105	11.7	4.92
B7ZBY6	*Ube2v1*	Ubiquitin-conjugating enzyme E2 variant 1 (Fragment)	m	8.0	7	1	1	1	125	13.9	5.25
Q9R1Z0	*Vdac3*	Voltage-dependent anion-selective channel protein 3	r	7.4	2	1	2	3	283	30.8	8.70
G3V8A5	*Vps35*	Vacuolar protein sorting-associated protein 35	r	4.2	2	3	3	3	796	91.7	5.44
Q5BJU7	*Wasf1*	Wiskott-Aldrich syndrome protein family member 1	r	4.1	2	2	2	3	559	61.5	6.37
Q9ESZ0	*Xrcc1*	DNA repair protein XRCC1	r	2.2	1	1	1	1	631	68.8	6.60
P63101	*Ywhaz*	14-3-3 protein zeta/delta	m	50.2	3	8	11	75	245	27.8	4.79
B1WBW3	*Zfp579*	Zinc finger protein 579	r	2.9	2	1	1	1	562	60.8	8.72
