# Supplementary material for: Comparative Proteomics Unveils LRRFIP1 as a New Player in the DAPK1 Interactome of Neurons Exposed to Oxygen and Glucose Deprivation
Source: Antioxidants (Basel). 2020 Nov 30;9(12):1202. doi: 10.3390/antiox9121202 (PMC7761126; doi:10.3390/antiox9121202)
Supplement: Supplementary file 1 [file antioxidants-09-01202-s001.zip › Table S2.pdf]

**Supplementary material** of the MS '**Comparative proteomics unveils LRRFIP1 as a new player in the DAPK1 interactome of neurons exposed to oxygen and glucose deprivation'**, by DeGregorio-Rocasolano et al.

**Table S2.** Protein partners in the DAPK1 interactome that are present only in neurons exposed to OGD (abbreviations: m: mouse; r: rat; Cp: Cavia porcellus; Cg: Cricetulus griseus; Ds: Dipus sagitta; Sp: Spermophilus parryii).

| Accession | Gene symbol | Description | Species | ΣCoverage | Σ# Proteins | Σ# Unique Peptides | Σ# Peptides | Σ# PSMs | # AAs | MW [kDa] | calc. pI |
|---|---|---|---|---|---|---|---|---|---|---|---|
| Q2LDP2 | - | Pyruvate dehydrogenase E1 alpha (Fragment) | Sp | 32.8 | 5 | 2 | 2 | 4 | 61 | 6.8 | 5.19 |
| P50475 | *Aars* | Alanine-tRNA ligase, cytoplasmic | r | 6.1 | 3 | 5 | 5 | 10 | 968 | 106.7 | 5.62 |
| P07872 | *Acox1* | Peroxisomal acyl-coenzyme A oxidase 1 | r | 1.4 | 1 | 1 | 1 | 1 | 661 | 74.6 | 8.47 |
| Q9D9S0 | *Acsl5* | Acyl-CoA synthetase long-chain family member 5, isoform CRA_a | m | 4.1 | 7 | 1 | 1 | 1 | 268 | 29.6 | 5.96 |
| G3V9U1 | *Agap1* | Centaurin, gamma 2 (Predicted) | r | 1.8 | 2 | 1 | 1 | 1 | 669 | 73.7 | 8.25 |
| Q8VHH5 | *Agap3* | Arf-GAP with GTPase, ANK repeat and PH domain-containing protein 3 | m | 1.5 | 1 | 1 | 1 | 3 | 910 | 97.9 | 7.75 |
| P0C6S7 | *Anks1b* | Ankyrin repeat and sterile alpha motif domain-containing protein 1B | r | 0.9 | 1 | 1 | 1 | 2 | 1260 | 139.1 | 6.30 |
| Q5XJY5 | *Arcn1* | Coatomer subunit delta | m | 2.2 | 2 | 1 | 1 | 1 | 511 | 57.2 | 6.21 |
| P07340 | *Atp1b1* | Sodium/potassium-transporting ATPase subunit beta-1 | r | 3.6 | 3 | 1 | 1 | 2 | 304 | 35.2 | 8.65 |
| G3V7L8 | *Atp6v1e1* | ATPase, H+ transporting, V1 subunit E isoform 1, isoform CRA_a | r | 3.5 | 3 | 1 | 1 | 1 | 226 | 26.1 | 8.43 |
| O35143 | *Atpif1* | ATPase inhibitor, mitochondrial | m | 10.4 | 2 | 1 | 1 | 1 | 106 | 12.2 | 9.64 |
| Q3TGG2 | *Atxn2l* | Ataxin-2-like protein | m | 1.7 | 2 | 1 | 1 | 2 | 994 | 105.4 | 7.97 |
| Q5HZI2 | *C2cd4c* | C2 calcium-dependent domain-containing protein 4C | m | 3.3 | 2 | 1 | 1 | 1 | 419 | 44.6 | 9.73 |
| Q8VDP4 | *Ccar2* | Cell cycle and apoptosis regulator protein 2 | m | 1.6 | 1 | 1 | 1 | 1 | 922 | 102.9 | 5.25 |
| D3ZZM9 | *Ccsap* | Centriole, cilia and spindle-associated protein | r | 4.8 | 1 | 1 | 1 | 1 | 252 | 28.4 | 9.23 |
| Q5FVI4 | *Cend1* | Cell cycle exit and neuronal differentiation protein 1 | r | 8.7 | 1 | 1 | 1 | 1 | 149 | 15.0 | 8.97 |
| Q9D9P1 | *Chchd3* | Coiled-coil-helix-coiled-coil-helix domain-containing protein 3, mitochondrial | m | 6.3 | 3 | 1 | 1 | 1 | 175 | 20.4 | 8.27 |
| D4A7N1 | *Chchd6* | Coiled-coil-helix-coiled-coil-helix domain-containing protein 6, mitochondrial | r | 7.7 | 2 | 2 | 2 | 2 | 261 | 29.2 | 7.94 |

| | | | | | | | | | | | |
|---|---|---|---|---|---|---|---|---|---|---|---|
| Q9D8B3 | *Chmp4b* | Charged multivesicular body protein 4b | m | 11.2 | 2 | 2 | 2 | 4 | 224 | 24.9 | 4.82 |
| Q6Q0N0 | *Clstn1* | Calsyntenin-1 | r | 0.7 | 2 | 1 | 1 | 1 | 952 | 106.2 | 5.01 |
| A2AI78 | *Cnksr2* | Connector enhancer of kinase suppressor of Ras 2 | m | 1.2 | 3 | 1 | 1 | 1 | 896 | 101.6 | 7.11 |
| P13233 | *Cnp* | 2',3'-cyclic-nucleotide 3'-phosphodiesterase | r | 3.1 | 1 | 1 | 1 | 1 | 420 | 47.2 | 8.90 |
| P00787 | *Ctsb* | Cathepsin B | r | 7.1 | 2 | 2 | 2 | 4 | 339 | 37.4 | 5.60 |
| P11030 | *Dbi* | Acyl-CoA-binding protein | r | 39.1 | 3 | 2 | 2 | 6 | 87 | 10.0 | 8.82 |
| Q6AY55 | *Dcakd* | Dephospho-CoA kinase domain-containing protein | r | 4.6 | 1 | 1 | 1 | 2 | 240 | 27.3 | 9.69 |
| Q641Y8 | *Ddx1* | ATP-dependent RNA helicase DDX1 | r | 3.4 | 2 | 2 | 2 | 2 | 740 | 82.4 | 7.23 |
| Q9WTM2 | *DDX6* | Probable ATP-dependent RNA helicase DDX6 (Fragment) | Cp | 4.5 | 3 | 2 | 2 | 2 | 472 | 53.2 | 8.66 |
| Q5RJY4 | *Dhrs7b* | Dehydrogenase/reductase SDR family member 7B | r | 5.2 | 1 | 1 | 1 | 1 | 325 | 35.3 | 9.55 |
| D4A9D6 | *Dhx9* | DEAH (Asp-Glu-Ala-His) box polypeptide 9 (Predicted) | r | 1.0 | 2 | 1 | 1 | 1 | 1174 | 131.6 | 6.35 |
| B1AR31 | *Dlg4* | Discs, large homolog 4 (Drosophila) (Fragment) | m | 4.8 | 3 | 1 | 1 | 1 | 248 | 26.5 | 5.00 |
| D4A9H1 | *Dlg5* | Discs, large homolog 5 (Drosophila) (Predicted) | r | 1.2 | 1 | 1 | 1 | 1 | 1355 | 150.8 | 8.02 |
| P63037 | *Dnaja1* | DnaJ homolog subfamily A member 1 | m | 10.3 | 4 | 3 | 3 | 7 | 397 | 44.8 | 7.08 |
| D4ABX4 | *Dnajb6* | DnaJ homolog subfamily B member 6 | r | 14.2 | 7 | 3 | 3 | 6 | 261 | 29.4 | 4.30 |
| Q8CIQ7 | *Dock3* | Dedicator of cytokinesis protein 3 | m | 0.6 | 1 | 1 | 1 | 1 | 2027 | 232.8 | 6.99 |
| D4A8N1 | *Dpm1* | Dolichol-phosphate mannosyltransferase subunit 1 | r | 4.6 | 1 | 1 | 1 | 1 | 260 | 29.2 | 9.50 |
| Q07139 | *Ect2* | Epithelial cell-transforming sequence 2 oncogene | m | 0.9 | 1 | 1 | 1 | 1 | 913 | 103.1 | 7.44 |
| Q3U5H6 | *Eif2s3x* | Tr-type G domain-containing protein (Fragment) | m | 4.2 | 4 | 1 | 1 | 1 | 330 | 35.7 | 8.54 |
| Q6NZJ6 | *Eif4g1* | Eukaryotic translation initiation factor 4 gamma 1 | m | 0.8 | 1 | 1 | 1 | 2 | 1600 | 176.0 | 5.40 |
| Q8VHU4 | *Elp1* | Elongator complex protein 1 | r | 1.8 | 2 | 2 | 2 | 2 | 1331 | 149.1 | 6.39 |
| Q8VEH5 | *Epm2aip1* | EPM2A-interacting protein 1 | m | 2.2 | 1 | 1 | 1 | 1 | 606 | 70.1 | 5.87 |
| Q6MG17 | *Flot1* | Flotillin 1 | r | 10.2 | 4 | 3 | 3 | 4 | 352 | 39.7 | 7.42 |
| Q5F258 | *Git1* | ARF GTPase-activating protein GIT1 | m | 1.8 | 3 | 1 | 1 | 1 | 761 | 84.1 | 6.74 |
| G3V9X2 | *Gpsm1* | G-protein signalling modulator 1 (AGS3-like, C. elegans), isoform CRA_c | r | 3.1 | 5 | 2 | 2 | 3 | 673 | 74.4 | 6.30 |
| A2AI19 | *Grin1* | Glutamate [NMDA] receptor subunit zeta-1 | m | 0.9 | 9 | 1 | 1 | 1 | 901 | 101.3 | 8.27 |
| G3V746 | *Grin2b* | Glutamate [NMDA] receptor subunit epsilon-2 | r | 1.0 | 4 | 1 | 1 | 1 | 1482 | 165.9 | 6.87 |
| P18266 | *Gsk3b* | Glycogen synthase kinase-3 beta | r | 2.6 | 3 | 1 | 1 | 1 | 420 | 46.7 | 8.78 |
| B8YDD1 | *Hax1* | HS1 binding protein variant IX | r | 8.0 | 2 | 1 | 1 | 3 | 151 | 17.1 | 6.21 |
| D4ADD3 | *Hecw2* | HECT, C2 and WW domain-containing E3 ubiquitin protein ligase 2 | r | 1.0 | 2 | 1 | 1 | 1 | 1578 | 176.4 | 5.39 |
| Q4U2R1 | *Herc2* | E3 ubiquitin-protein ligase HERC2 | m | 0.3 | 1 | 1 | 1 | 2 | 4836 | 527.1 | 6.27 |
| O54792 | *Hes2* | Transcription factor HES-2 | m | 5.1 | 1 | 1 | 1 | 1 | 157 | 17.2 | 10.07 |

| | | | | | | | | | | | |
|---|---|---|---|---|---|---|---|---|---|---|---|
| P13704 | *HMGCS1* | Hydroxymethylglutaryl-CoA synthase, cytoplasmic | Cg | 2.5 | 3 | 1 | 1 | 1 | 520 | 57.3 | 5.66 |
| D3ZC55 | *Hspa12a* | Heat shock 70kDa protein 12A (Predicted), isoform CRA_a | r | 4.0 | 1 | 2 | 2 | 3 | 675 | 74.8 | 6.61 |
| Q80Z01 | *Hspb3* | Small heat shock protein B3 (Fragment) | Ds | 5.3 | 2 | 1 | 1 | 1 | 131 | 14.5 | 5.27 |
| Q3KR86 | *Immt* | Mitochondrial inner membrane protein (Fragment) | r | 8.2 | 2 | 2 | 4 | 7 | 609 | 67.1 | 5.80 |
| Q8CAQ8 | *Immt* | Mitochondrial inner membrane protein | m | 4.6 | 2 | 1 | 3 | 3 | 757 | 83.8 | 6.61 |
| D3ZLZ7 | *Impdh1* | Inosine-5'-monophosphate dehydrogenase 1 | r | 2.3 | 2 | 1 | 1 | 1 | 514 | 55.3 | 6.80 |
| E9PU28 | *Impdh2* | Inosine-5'-monophosphate dehydrogenase 2 | r | 2.3 | 3 | 1 | 1 | 3 | 514 | 55.8 | 7.28 |
| D3YUD3 | *Inpp4a* | Type I inositol 3,4-bisphosphate 4-phosphatase | m | 1.2 | 3 | 1 | 1 | 1 | 939 | 105.5 | 6.98 |
| Q7TQK1 | *Ints7* | Integrator complex subunit 7 | m | 1.5 | 1 | 1 | 1 | 1 | 966 | 106.8 | 8.22 |
| B2KF90 | *Itpr2* | Inositol 1,4,5-triphosphate receptor 2 | m | 0.4 | 3 | 1 | 1 | 1 | 2668 | 303.8 | 6.47 |
| Q3YAA9 | *Kcnip3* | Calsenilin isoform 4 | m | 3.5 | 4 | 1 | 1 | 1 | 230 | 26.4 | 4.77 |
| B1AQZ5 | *Kif3a* | Kinesin family member 3A (Fragment) | m | 6.0 | 5 | 1 | 1 | 1 | 185 | 21.8 | 8.03 |
| Q9ERE2 | *Krt81* | Keratin, type II cuticular Hb1 (Fragment) | m | 2.8 | 5 | 1 | 1 | 2 | 390 | 43.7 | 5.20 |
| Q9Z2T6 | *Krt85* | Keratin, type II cuticular Hb5 | m | 2.0 | 1 | 1 | 1 | 1 | 507 | 55.7 | 6.42 |
| Q8K310 | *Matr3* | Matrin-3 | m | 7.6 | 3 | 5 | 5 | 8 | 846 | 94.6 | 6.25 |
| Q8BI84 | *Mia3* | Melanoma inhibitory activity protein 3 | m | 0.7 | 1 | 1 | 1 | 1 | 1930 | 213.5 | 4.75 |
| Q5M9I6 | *Mmtag2* | Multiple myeloma tumor-associated protein 2 homolog | r | 3.1 | 2 | 1 | 1 | 1 | 260 | 29.3 | 9.85 |
| D3Z9R8 | *Mp68* | 6.8 kDa mitochondrial proteolipid | r | 18.3 | 1 | 1 | 1 | 1 | 60 | 6.9 | 9.92 |
| Q8CH77 | *Nav1* | Neuron navigator 1 | m | 3.9 | 1 | 6 | 6 | 9 | 1875 | 202.2 | 8.06 |
| Q9Z0W3 | *Nup160* | Nuclear pore complex protein Nup160 | m | 0.7 | 1 | 1 | 1 | 2 | 1402 | 158.1 | 5.52 |
| A0JPJ7 | *Ola1* | Obg-like ATPase 1 | r | 10.4 | 3 | 3 | 3 | 6 | 396 | 44.5 | 7.77 |
| G3V9G0 | *Pcdhb21* | Protocadherin beta 21 | r | 2.5 | 1 | 1 | 1 | 1 | 757 | 82.8 | 4.79 |
| P31044 | *Pebp1* | Phosphatidylethanolamine-binding protein 1 | r | 14.4 | 2 | 2 | 2 | 7 | 187 | 20.8 | 5.80 |
| Q9DBD5 | *Pelp1* | Proline-, glutamic acid- and leucine-rich protein 1 | m | 1.4 | 2 | 1 | 1 | 1 | 1123 | 118.0 | 4.36 |
| Q9EPC6 | *Pfn2* | Profilin-2 | r | 10.0 | 2 | 1 | 1 | 4 | 140 | 15.0 | 6.99 |
| Q7TQG1 | *Plekha6* | Pleckstrin homology domain-containing family A member 6 | m | 1.0 | 1 | 1 | 1 | 1 | 1173 | 131.3 | 8.97 |
| P37230 | *Ppara* | Peroxisome proliferator-activated receptor alpha | r | 2.6 | 1 | 1 | 1 | 1 | 468 | 52.3 | 6.25 |
| P60469 | *Ppfia3* | Liprin-alpha-3 | m | 1.0 | 3 | 1 | 1 | 1 | 1043 | 116.2 | 5.96 |
| Q80Y24 | *Prickle2* | Prickle-like protein 2 | m | 1.1 | 2 | 1 | 1 | 1 | 845 | 95.7 | 7.27 |
| P68404 | *Prkcb* | Protein kinase C beta type | m | 5.2 | 4 | 3 | 3 | 3 | 671 | 76.7 | 7.01 |
| P09216 | *Prkce* | Protein kinase C epsilon type | r | 7.3 | 2 | 4 | 4 | 4 | 737 | 83.4 | 6.95 |
| O08618 | *Prpsap2* | Phosphoribosyl pyrophosphate synthase-associated protein 2 | r | 2.4 | 2 | 1 | 1 | 2 | 369 | 40.8 | 7.17 |
| Q2PFD7 | *Psd3* | PH and SEC7 domain-containing protein 3 | m | 2.2 | 2 | 2 | 2 | 2 | 1037 | 114.7 | 6.24 |
| P62192 | *Psmc1* | 26S protease regulatory subunit 4 | m | 5.5 | 1 | 1 | 2 | 2 | 440 | 49.2 | 6.21 |

| | | | | | | | | | | | |
|---|---|---|---|---|---|---|---|---|---|---|---|
| G3V7L6 | *Psmc2* | 26S protease regulatory subunit 7 | r | 8.6 | 4 | 3 | 3 | 4 | 433 | 48.6 | 5.95 |
| P62334 | *Psmc6* | 26S protease regulatory subunit 10B | m | 3.1 | 2 | 1 | 1 | 2 | 389 | 44.1 | 7.49 |
| F1LMZ8 | *Psmd11* | 26S proteasome non-ATPase regulatory subunit 11 | r | 15.2 | 3 | 5 | 5 | 7 | 422 | 47.4 | 6.48 |
| O35226 | *Psmd4* | 26S proteasome non-ATPase regulatory subunit 4 | m | 4.0 | 3 | 1 | 1 | 2 | 376 | 40.7 | 4.79 |
| Q3UEB3 | *Puf60* | Poly(U)-binding-splicing factor PUF60 | m | 1.8 | 1 | 1 | 1 | 1 | 564 | 60.2 | 5.29 |
| G3X8R5 | *Qrich1* | Glutamine-rich protein 1 | m | 1.2 | 2 | 1 | 1 | 1 | 777 | 86.5 | 5.87 |
| P0C643 | *Rasgrp2* | RAS guanyl-releasing protein 2 | r | 2.3 | 2 | 1 | 1 | 1 | 608 | 69.2 | 7.68 |
| Q3TTW9 | *Rgs12* | Regulator of G-protein-signaling 12 | m | 2.8 | 5 | 1 | 1 | 1 | 499 | 54.8 | 8.65 |
| Q91YL2 | *Rnf126* | RING finger protein 126 | m | 5.4 | 2 | 1 | 1 | 1 | 313 | 34.1 | 5.17 |
| P24049 | *Rpl17* | 60S ribosomal protein L17 | r | 7.6 | 3 | 1 | 1 | 2 | 184 | 21.4 | 10.18 |
| D4AAZ6 | *Rpl37a* | 60S ribosomal protein L37a | r | 16.7 | 2 | 1 | 1 | 1 | 72 | 8.1 | 10.37 |
| Q9JKY0 | *Rqcd1* | Cell differentiation protein RCD1 homolog | m | 3.7 | 1 | 1 | 1 | 1 | 299 | 33.6 | 8.03 |
| P60122 | *Ruvbl1* | RuvB-like 1 | m | 5.9 | 1 | 2 | 2 | 3 | 456 | 50.2 | 6.42 |
| O08804 | *Serpinb6b* | Serine (or cysteine) peptidase inhibitor, clade B, member 6B | m | 4.0 | 1 | 1 | 1 | 4 | 377 | 42.5 | 5.26 |
| A0JLN0 | *Sf3b1* | Splicing factor 3b, subunit 1 (Fragment) | m | 3.8 | 3 | 1 | 1 | 1 | 496 | 54.4 | 6.10 |
| Q8BXU5 | *Sh3gl2* | Endophilin-A1 | m | 2.9 | 6 | 1 | 1 | 1 | 245 | 27.9 | 5.77 |
| D3YZU1 | *Shank1* | SH3 and multiple ankyrin repeat domains protein 1 | m | 0.7 | 2 | 1 | 1 | 1 | 2167 | 226.2 | 8.34 |
| Q5FVG4 | *Slc25a22* | Solute carrier family 25 (Mitochondrial carrier, glutamate), member 22 | r | 3.9 | 3 | 1 | 1 | 1 | 229 | 24.6 | 8.60 |
| O35413 | *Sorbs2* | Sorbin and SH3 domain-containing protein 2 | r | 0.8 | 1 | 1 | 1 | 3 | 1196 | 134.0 | 8.46 |
| O08623 | *Sqstm1* | Sequestosome-1 | r | 3.6 | 1 | 1 | 1 | 1 | 439 | 47.7 | 5.17 |
| Q3TWL2 | *Tmem55b* | Transmembrane protein 55B | m | 7.4 | 4 | 1 | 2 | 2 | 284 | 30.0 | 8.82 |
| Q3TC52 | *Trim46* | Tripartite motif-containing protein 46 | m | 2.0 | 2 | 1 | 1 | 1 | 541 | 60.7 | 8.63 |
| Q8BJG7 | *Ttll1* | Probable tubulin polyglutamylase TTLL1 | m | 4.0 | 3 | 1 | 1 | 2 | 297 | 34.9 | 9.26 |
| P83887 | *Tubg1* | Tubulin gamma-1 chain | m | 2.0 | 2 | 1 | 1 | 1 | 451 | 51.1 | 6.02 |
| Q6P5E4 | *Uggt1* | UDP-glucose:glycoprotein glucosyltransferase 1 | m | 0.8 | 2 | 1 | 1 | 1 | 1551 | 176.3 | 5.62 |
| Q68FY0 | *Uqcrc1* | Cytochrome b-c1 complex subunit 1, mitochondrial | r | 4.6 | 2 | 2 | 2 | 4 | 480 | 52.8 | 5.88 |
| D3ZC84 | *Usp9x* | Ubiquitin carboxyl-terminal hydrolase | r | 1.9 | 3 | 4 | 4 | 6 | 2547 | 289.3 | 5.78 |
| Q9WV55 | *Vapa* | Vesicle-associated membrane protein-associated protein A | m | 10.4 | 5 | 1 | 2 | 2 | 249 | 27.8 | 8.40 |
| O08700 | *Vps45* | Vacuolar protein sorting-associated protein 45 | r | 2.1 | 3 | 1 | 1 | 1 | 570 | 64.9 | 8.25 |
| Q9ERH3 | *Wdr7* | WD repeat-containing protein 7 | r | 2.8 | 3 | 3 | 3 | 5 | 1488 | 163.1 | 7.02 |
| Q4QR85 | *Wdr77* | Methylosome protein 50 | r | 4.4 | 2 | 1 | 1 | 1 | 342 | 37.1 | 5.27 |
| Q6NXJ0 | *Wwc2* | WW domain-containing protein 2 | m | 1.5 | 2 | 1 | 1 | 1 | 1187 | 132.5 | 5.71 |
| F1LM93 | *Yes1* | Tyrosine-protein kinase Yes | r | 2.2 | 3 | 1 | 1 | 1 | 541 | 60.6 | 6.64 |
